# Strategies and approaches to vector control effectiveness toward the elimination of malaria in mainland Tanzania: An implementation case study analysis

**DOI:** 10.1371/journal.pgph.0005574

**Published:** 2026-10-22

**Authors:** Mponeja Peter Gitanya, Matt Worges, Hannah Koenker, Samson Kiware, Samwel Lazaro, Jovin Kitau, William Kisinza, Naomi Serbantez, Benjamin Kamala, Charles Dismas, Johnson Matowo

**Affiliations:** 1 Tanzania National Malaria Control Programme, Ministry of Health, Dodoma, Tanzania; 2 KCMC University, Kilimanjaro, Tanzania; 3 PMI Tanzania Vector Control Activity, Tropical Health Limited Liability Partnership, Tel Aviv, Israel; 4 PMI Tanzania Vector Control Activity, Tropical Health Limited Liability Partnership, Baltimore, Maryland, United States of America; 5 Tanzania Ifakara Health Institute, Dar es Salaam, Tanzania; 6 World Health Organization Tanzania Country Office, Dar es Salaam, Tanzania; 7 National Institute for Medical Research, Amani Research Centre, Tanga, Tanzania; 8 U.S. President’s Malaria Initiative, USAID, Dar es Salaam, Tanzania; 9 School of Public Health and Social Sciences, Muhimbili University of Health and Allied Sciences, Dar es Salaam, Tanzania; SUPA71 Co., Ltd, THAILAND

## Abstract

Effective malaria control and elimination require continuous monitoring of vector control interventions, entomological indicators, and their relationship with malaria case incidence. This implementation case study describes data fragmentation and develops a unified, granular profile of malaria burden and vector control at the district level in mainland Tanzania. By integrating data on malaria burden, vector control interventions, and intervention coverage, the study complements existing stratification approaches and generates actionable insights to inform policy and strategic decision-making on effective interventions at the council level. We extracted monthly council-level malaria incidence data (2018–2022) from DHIS2. Insecticide-treated net (ITN) access was estimated using ITN distribution and population data through a stock-and-flow model, assuming a Tanzania-specific median net lifespan of 2.15 years. Data on indoor residual spraying (IRS) implementation and entomological monitoring reports (2011–2022) were reviewed. Rainfall data were summarized and visualized as overlapping monthly time series. According to the annual vector control report (AVCR) for 2021 and 2022, the mean modeled council-level population access to ITNs was 63.9% in 2021 and 65.2% in 2022. Across 184 councils, 17.4% had low ITN access (<50%), 59.2% had moderate access (51–80%), and 23.4% achieved sufficient access (>80%). Councils with ITN access above 80% consistently reported lower malaria incidence rates, whereas those with access below 50% were generally associated with higher malaria burden. Higher ITN access was associated with lower malaria incidence, underscoring the importance of accurate quantification and timely distribution of insecticide-treated nets. In areas where *Anopheles arabiensis* predominates, complementary outdoor vector control interventions may be needed to address residual outdoor transmission. District-level vector control profiling can enhance the effectiveness of malaria programmes by supporting evidence-based resource allocation and adaptive intervention strategies.

## Introduction

Malaria remains a significant public health challenge. Globally, malaria cases increased by 11 million, from 249 million in 2022 to an estimated 263 million in 2023 [1]. The World Health Organization (WHO) African region is disproportionately affected, accounting for 94% of global malaria cases in 2023. Tanzania alone contributed to 4% of global malaria deaths in 2022, with nearly 95% of its mainland population at risk of the disease [2]. Malaria control and elimination efforts rely heavily on insecticide-based vector control interventions. Recent success in malaria control in sub-Saharan African countries, including Tanzania, is mainly attributed to the scale-up of insecticide-treated nets (ITNs) and indoor residual spraying (IRS) interventions [3].

In sub-Saharan countries, including East African countries, the incidence of malaria cases has continued to rise due to various factors, including climate [4,5], despite the deployment of malaria control interventions [1]. To reduce the malaria burden, Tanzania, like other malaria-endemic countries such as Kenya and Uganda, has stratified its intervention packages and strategies according to malaria transmission intensity [6–8]. Interventions are deployed using a mixed-methods map tailored to local cultural contexts at the subnational level (councils), for both preventive and curative interventions, to maximize impact [2,9]. Given funding constraints, a targeted strategy should be considered essential to sustain malaria reductions and prevent resurgence in endemic countries, while supporting progress toward national malaria control and elimination targets [10].

The intersection between environmental change and malaria—specifically, climate variability, including changes in temperature, rainfall, and altitude—has affected the abundance, behavior, distribution, breeding patterns, ecology, and survival of malaria vector mosquitoes in Tanzania [2,11,12]. Temperature and precipitation show unimodal relationships with malaria: a positive effect up to a point, followed by an adverse effect as these variables increase, as shown by shifts in malaria distribution and trends in countries such as Zimbabwe and Ethiopia [5,13,14]. Temperature changes from climate change can shift malaria’s geographic range; thus, malaria-free areas may become vulnerable to transmission, potentially leading to a resurgence in places where it was previously under control [15]. Malaria transmission is also influenced by a combination of environmental, geographic, socioeconomic, and epidemiological factors, including vegetation density [16].

Moreover, climate change can indirectly influence malaria risk by contributing to malnutrition, which is a recognized risk factor for severe malaria, particularly among young children and pregnant women. Extreme weather events such as heatwaves and flooding may increase disease transmission and burden, leading to population displacement as more people without immunity migrate to endemic areas [17]. Vulnerable populations, such as those living in poverty, may be disproportionately affected by the impacts of climate change on malaria. Moreover, in the lowlands of malaria-endemic western Kenya, variations in climatic factors played a key role in driving malaria incidence during 2008–2019, despite high bed net coverage and use [5,14–16,18–22].

Climate change is just one of many threats to the global malaria control and elimination efforts. At the same time, persistent gaps in investment and programme support have limited access to the services needed to prevent, detect, and treat malaria. Together, these challenges continue to hamper progress toward malaria control and elimination globally and in Tanzania [5,23].

Tanzania has made significant progress in scaling up control strategies, including vector control and malaria case management interventions. The overall malaria incidence rate per 1,000 at-risk population has decreased from 118 cases in 2018–58 cases in 2022 [24]. Despite this achievement, further progress has stalled; for example, malaria prevalence among children under 5 years of age increased from 7.5% in 2017 to 8.1% in 2022. Additionally, regional disparities persist across the country and between sociodemographic and socioeconomic groups [25,26].

Since the early 2000s, Tanzania has implemented two core malaria vector control interventions: ITN distribution and IRS. ITN distribution has been universal via multiple delivery mechanisms [2], including mass campaigns, the school net program (SNP), antenatal clinics (ANC), and the expanded program for immunization (EPI). IRS implementation was limited to a small number of councils [2]. Meanwhile, larval source management (LSM), i.e., biolarviciding and source reduction, has been used as a supplementary strategy alongside ITNs or IRS, particularly in areas where breeding sites are few, fixed, and readily identifiable [2,27].

The major malaria vectors in mainland Tanzania have been members of the *Anopheles funestus* group and the *Anopheles gambiae* complex [2]. Several pieces of evidence, including data from the Tanzania longitudinal entomological surveillance, indicate the predominance of *Anopheles arabiensis*, a primarily outdoor-feeding species of the *Anopheles gambiae* complex and *Anopheles funestus* [11,28]. Despite the successes of core vector control interventions, as seen in other endemic areas, the emergence and spread of insecticide-resistant strains, behavioral avoidance of insecticide-based tools [29–38], and a shift toward exophagic (outdoor feeding) and exophilic (outdoor resting) behaviors are threatening to undermine the gains made so far [33,39,40].

The increased heterogeneity in malaria transmission and disease burden necessitated the country’s stratification of malaria risk according to epidemiological diversity. Malaria stratification marked a strategic shift from a “one-size-fits-all” approach to a more tailored approach to malaria control, optimizing resources and enhancing impact [41,42]. The stratification produced four epidemiological classes—“very low,” “low,” “moderate,” and “high” burden strata—and one operational stratum, “urban.” Areas of moderate-to-high transmission have since been targeted for burden reduction, while very low- and low-transmission areas have been targeted for elimination [41]. To strengthen Tanzania’s health system, malaria risk stratification necessitated elevating malaria surveillance to a core intervention, critical to malaria elimination programs [42–44]. This occurred in parallel with efforts to strengthen data systems to inform national and subnational malaria strategies and support periodic updates to the stratification [9].

Consequently, in Tanzania, malaria stratification aligns with integrated vector management by tailoring vector control interventions to local transmission settings. While malaria stratification in most countries relies primarily on epidemiological indicators, Tanzania has expanded this approach by incorporating entomological indicators from the annual vector control profile (AVCR) into district-level stratification [45]. Data from multiple fragmented sources, ranging from ITN distribution records to entomological surveillance, made it challenging to create a unified picture at the council level, and the impacts of this data were not well articulated. Moreover, the lack of integration also hindered the ability of the National Malaria Control Programme (NMCP) to tailor vector control interventions to local epidemiological data and to make data-driven decisions at the subnational level [2,46]. Most household surveys provide only regional estimates, limiting the granularity needed for targeted interventions at the council level.

To address these challenges, this case study synthesized and documented a comprehensive Annual Vector Control Report (AVCR) for mainland Tanzania, drawing on multiple data sources and presenting findings through council-level profiles that provide a detailed, localized overview of malaria burden, vector control interventions, and entomological indicators [45]. By integrating intervention coverage data into district-level stratification, the AVCR becomes a stronger decision-making tool. The report is intended to help the NMCP and subnational authorities translate data into action, particularly by improving intervention coverage in the most affected areas and supporting more precise, evidence-based decisions tailored to local transmission dynamics. This approach helps ensure that high-transmission councils are prioritized for intensified interventions, while areas with stable, low malaria prevalence are not over-resourced. Furthermore, the AVCR supports a more dynamic and adaptive malaria control strategy by enabling timely adjustments to interventions in response to emerging challenges, such as insecticide resistance, vector behavioral changes, and shifts in malaria incidence.

## Materials and implementation methods

### Ethics statement

KCMC University in Kilimanjaro, Moshi, Tanzania, granted research ethical clearance certificate number 2667.

The AVCR compiled and harmonized data from 184 councils across all 26 regions of mainland Tanzania to support detailed council-level analysis and visualization. Key data sources included ITN distribution records from various channels spanning 2004–2022, malaria case incidence data from District Health Information System 2 (DHIS2) covering 2015–2023, and entomological surveillance reports from 2011 to 2021. Additional data sources included rainfall estimates from the Climate Hazards Group Infrared Precipitation with Stations (CHIRPS) dataset for 2015–2021 and population estimates from the 2012 National Population and Housing Census conducted by the Tanzania National Bureau of Statistics.

### Malaria case incidence analysis

Monthly confirmed malaria case data from 2018 to 2023 were extracted from the Tanzania DHIS2. The annual malaria case data from 2015 to 2017 were collated from the NMCP Malaria Surveillance Bulletins [24]. Malaria case counts were converted to incidence rates per 1,000 population using population estimates from the Tanzania National Bureau of Statistics [47]. Case incidence values were presented as monthly averages, year-over-year change, and monthly time series. Trends were analyzed using linear regression to classify councils as having increasing, decreasing, or stable malaria incidence. The slope of the linear best-fit line was considered not statistically different from zero if the associated *p-*value was less than 0.05. Where applicable, IRS and mass replacement campaign (MRC) implementation timelines were overlaid on incidence trends.

### Insecticide-treated net data sources

Historical ITN distribution data were compiled from different sources, including the Tanzania National Voucher Scheme (TNVS, 2004–2014), the under-five catch-up campaign (U5CC, 2007–2008), the universal coverage campaign (UCC, 2010–2011), the MRCs of years 2015 and 2020, routine channels through reproductive and child health clinics (RCH) during ANCs, the EPI from 2016 to 2022, and the SNP from 2013 to 2022. A stock-and-flow model was applied to estimate the annual net crop of surviving ITNs using a Tanzania-specific median net lifespan of 2.15 years (CI: 1.88–2.43) [3,48,49]. This was reliable data, supported by several national surveys, the physical durability of nets, community engagement, and cultural notions regarding net use [50]. The total net crop for each council was divided by the projected population, as reported in the 2012 National Bureau of Statistics census, to calculate nets per capita (NPC) per year, then converted to ITN access estimates using a nonparametric model derived from the Demographic Health Survey, phone surveys, school malaria parasitemia surveys, and Malaria Indicator Survey data [25,26,51,52]. The analysis also examined the proportion of nets issued by each channel, expressed as the number of “nets issued as a percentage of the council population.”

### Entomological surveillance and insecticide resistance

Entomological data, although limited to certain councils, included species composition, vector abundance, and insecticide resistance profiles, as reported in the entomological surveillance profile (2016–2021) [11]. The probability of species occurrence per council was derived from spatial modeling of entomological data from the 2012–2014 Malaria Atlas Project. Councils with documented resistance to any of the three pyrethroids (permethrin, deltamethrin, and alphacypermethrin) used in ITNs in Tanzania over three consecutive years were designated as having pyrethroid resistance. These data-informed recommendations are for ITN types and IRS deployment.

### Rainfall and seasonality

Monthly rainfall data were compiled at the council level from the Climate Hazards Group Infrared Precipitation with Stations (CHIRPS) dataset [53] for seven years (2015–2021) and presented as overlapping monthly time series relative to malaria case incidence trends as shown in Fig 1. Annual rainfall and malaria case incidence across all regions of Tanzania fall into two categories: unimodal and bimodal. In most rural councils, malaria transmission peaks immediately after each rainy season, whereas in urban councils (town councils, municipalities, and cities), the rainy season has little or no impact on malaria cases. As a result, these areas have lower overall transmission rates, along with improved housing, higher socioeconomic status (SES), and proximity to health clinics, all of which facilitate access to care and treatment.

**Fig 1:**
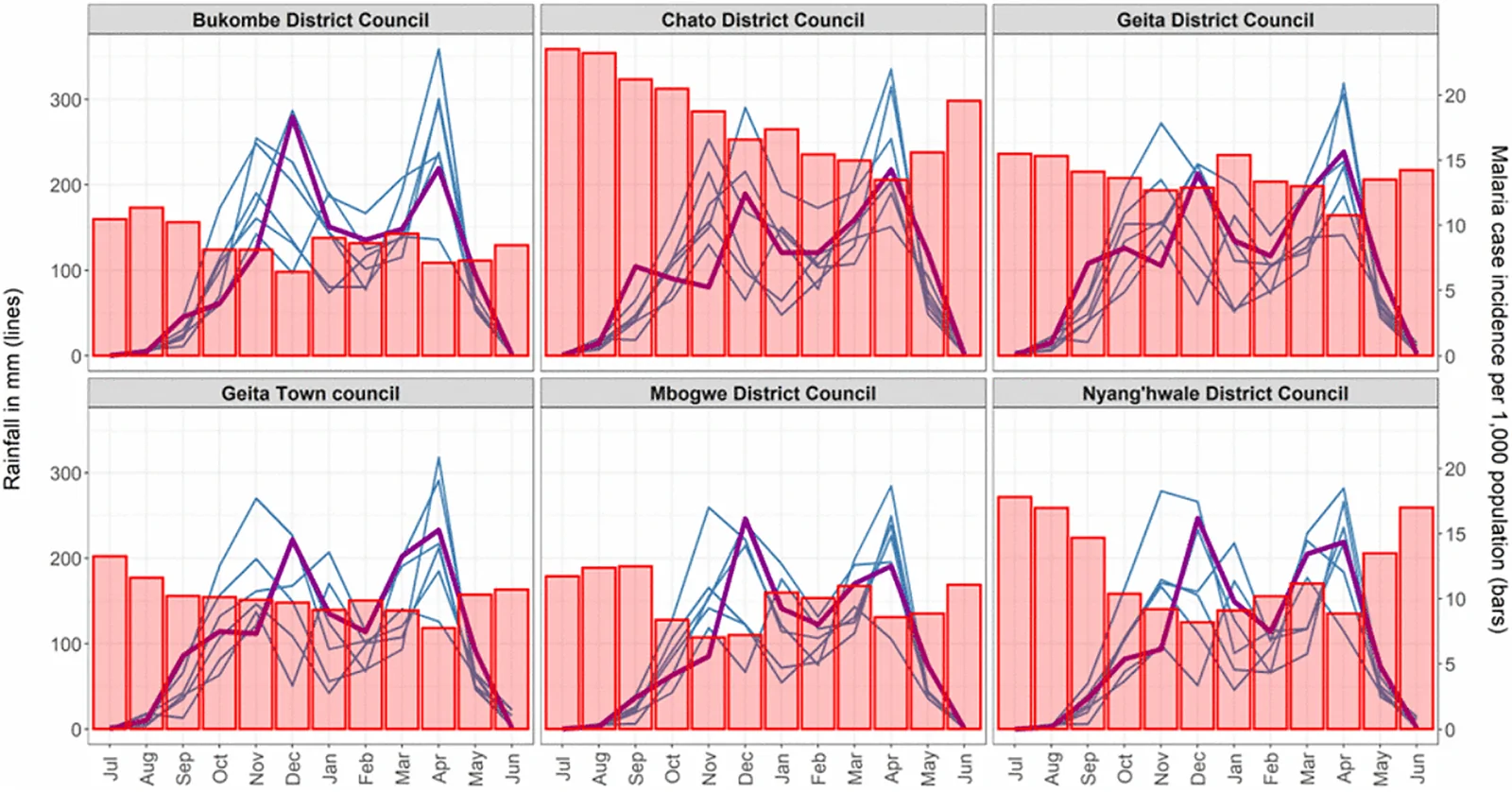
Total monthly rainfall and average monthly malaria case incidence.

### Council classification framework

Each council was categorized by ITN access levels (<50%, 51–80%, > 80%), confirmed malaria incidence trends (steady, increasing, or decreasing), and the presence of insecticide resistance. The results were presented as council-level maps by region. Councils were categorized based on whether “current interventions appear sufficient,” “current interventions are sufficient but increased coverage is needed,” or “new/additional interventions are needed to decrease malaria case incidence.” In councils with high coverage but persistent or rising malaria levels, especially those with a moderate-to-high burden, we recommended switching to dual-Active Ingredient (dual-AI) ITNs or IRS. Piperonyl butoxide (PBO) nets were recommended in councils with pyrethroid resistance, provided budget constraints did not compromise overall ITN coverage. Moreover, the PBO nets were quantified annually using the Roll Back Malaria initiative’s population quantifier to sustain ITN access at an optimal 80% of the population. For example, ITNs for mass campaigns every three years, calculated at one net per 1.8 people, are complemented by subsequent annual ITNs, as needed in Tanzania, through continuous ITN distribution mechanisms between mass campaigns [54].

## Results

### Vector control coverage

IRS has been conducted since 2009 in 25 councils across five high-malaria-burden regions, namely Geita, Kagera, Mwanza, Mara, and Kigoma, with some councils receiving up to 6 spraying rounds through 2022. Moreover, council coverage of IRS was above 90%, and it used neonicotinoid, pyrethroid, and organophosphate insecticide classes, selecting them based on expert recommendations and evidence of pyrethroid resistance.

In addition, Fig 2 illustrates the number of ITNs distributed in the Mara region through various delivery channels, including the Tanzania National Voucher Scheme (TNVS), Under-Five Catch-up Campaign (U5CC), Universal Coverage Campaign (UCC), Mass Replacement Campaign (MRC), School Net Programme (SNP), and Reproductive and Child Health (RCH) clinics (RCH). Before 2014, the TNVS was the only net distribution channel. However, the spikes observed in Fig 2 reflect MRC implementation, followed by annual SNP distributions between campaigns and the continuous delivery of ITNs through RCH services.

**Fig 2:**
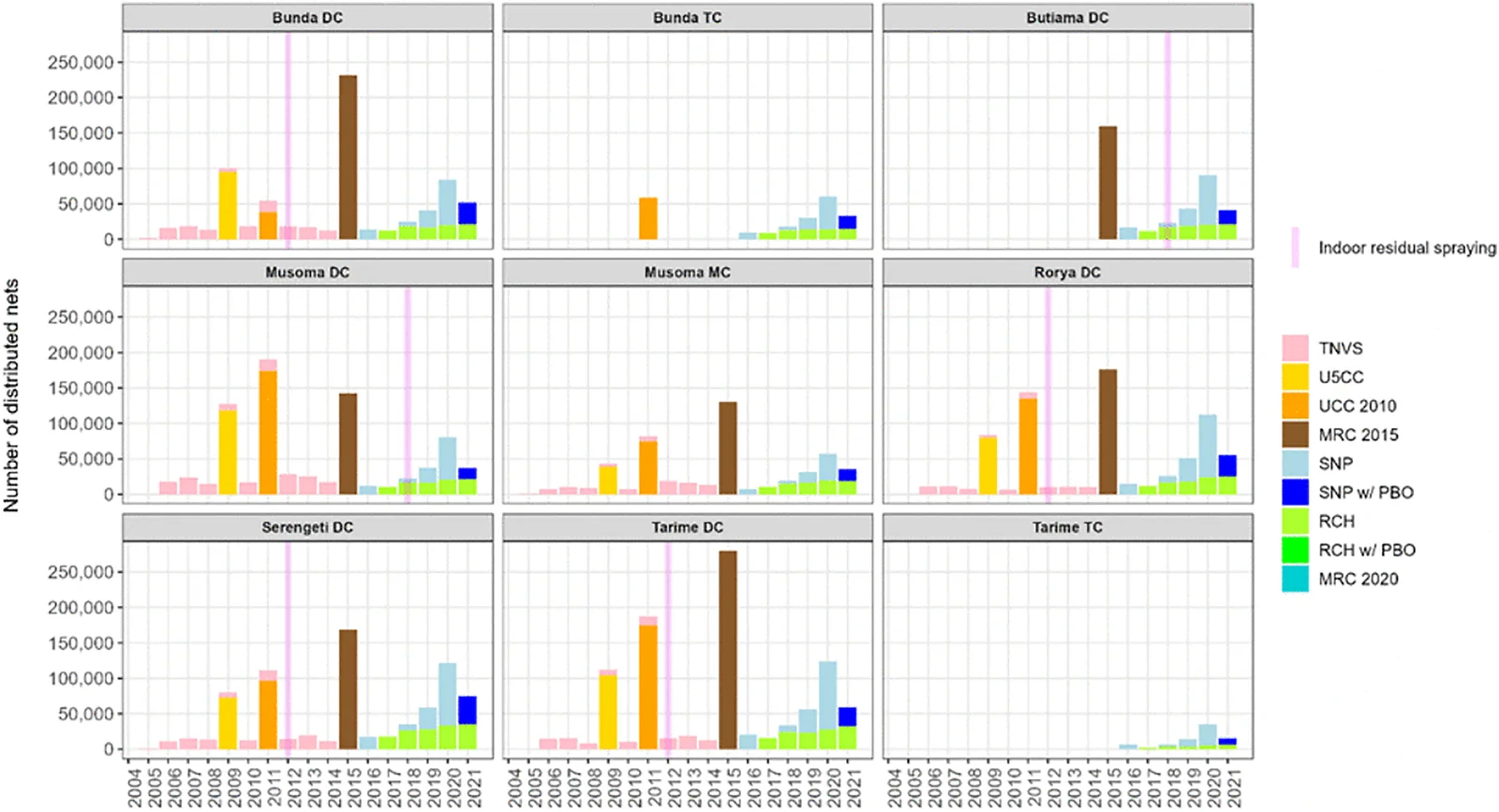
Bed net distribution by channel, year, and council with the IRS spray year.

The net crop is the sum of all net cohorts of annual distributions, carried forward and reduced by an annual loss rate defined by a loss function, as expressed in the Mwanza region (Fig 3).

**Fig 3:**
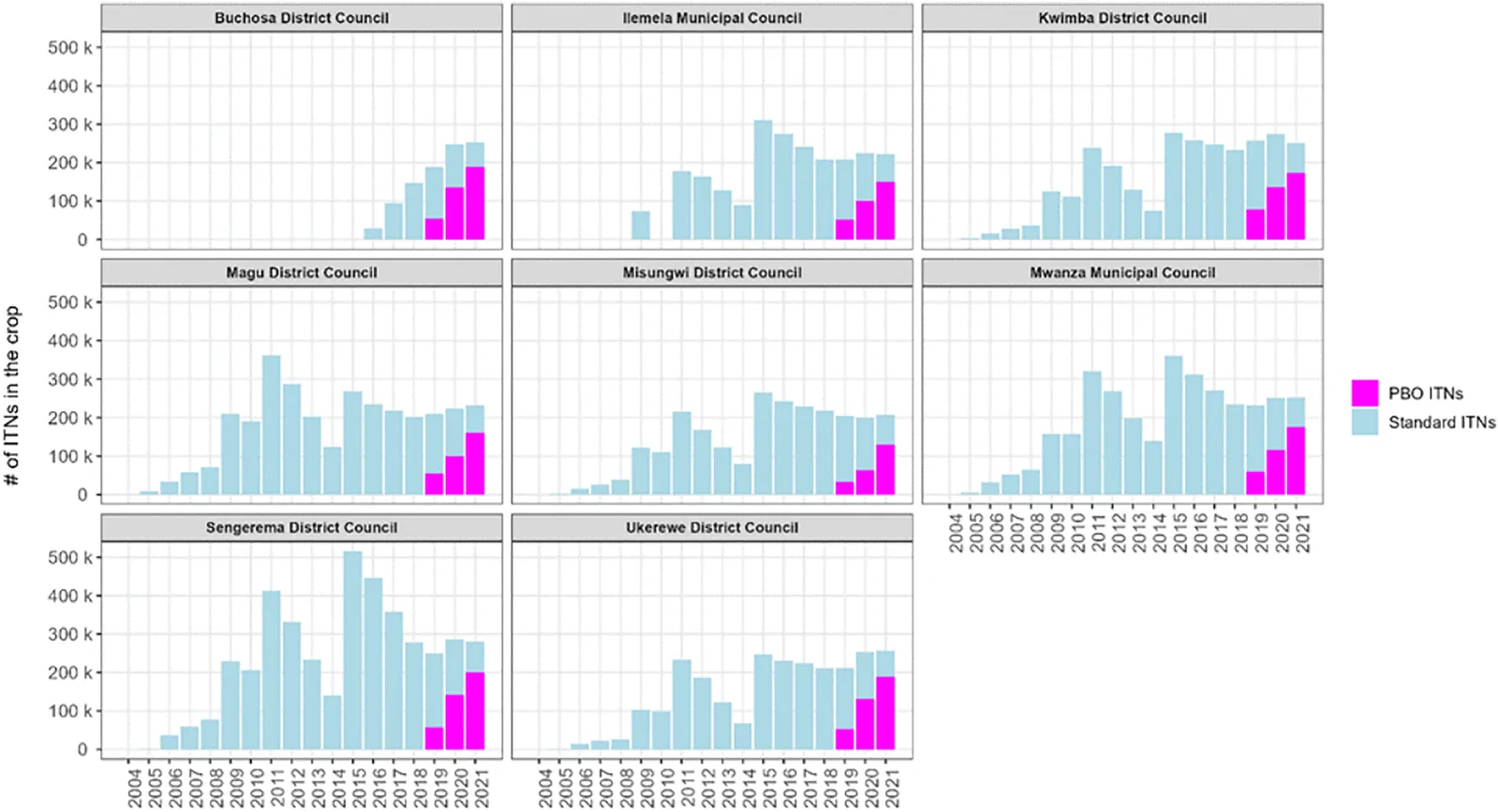
The net crop for PBO and standard ITNs by year, channel, and councils.

### ITN access trend and annual malaria case incidence

The Annual Vector Control Reports for 2021 and 2022 estimated mean council-level population access to ITNs at 63.9% and 65.2%, respectively. Across the three levels of population access to ITNs, 17.4% (32 of 184) of councils fell into the low range (<50%), 59.2% (109 of 184) fell into the moderate range (51–80%), and 23.4% (43 of 184) fell into the sufficient range (>80%) [45]. The percentage of councils in the low range nearly doubled, while the rate of councils in the sufficient range almost quintupled from 2021. The SNP maintained ITN access in participating councils, whereas areas relying exclusively on mass campaigns declined over time due to net attrition. Councils with ITN access above 80% consistently reported lower malaria incidence rates, while regions with low ITN access (<50%) often had a higher malaria burden, particularly in southern areas such as Morogoro, Mtwara, and Pwani.

For example, in the Geita region, population access to ITNs was moderate, averaging 73% across all councils in 2022, ranging from 64% in Nyang’hwale council to 78% in Chato council (Fig 4), and has leveled off since about 2020. Only Bukombe council was included in this region’s last IRS spray round, conducted in 2021, which used Actellic 300 CS (an organophosphate).

**Fig 4:**
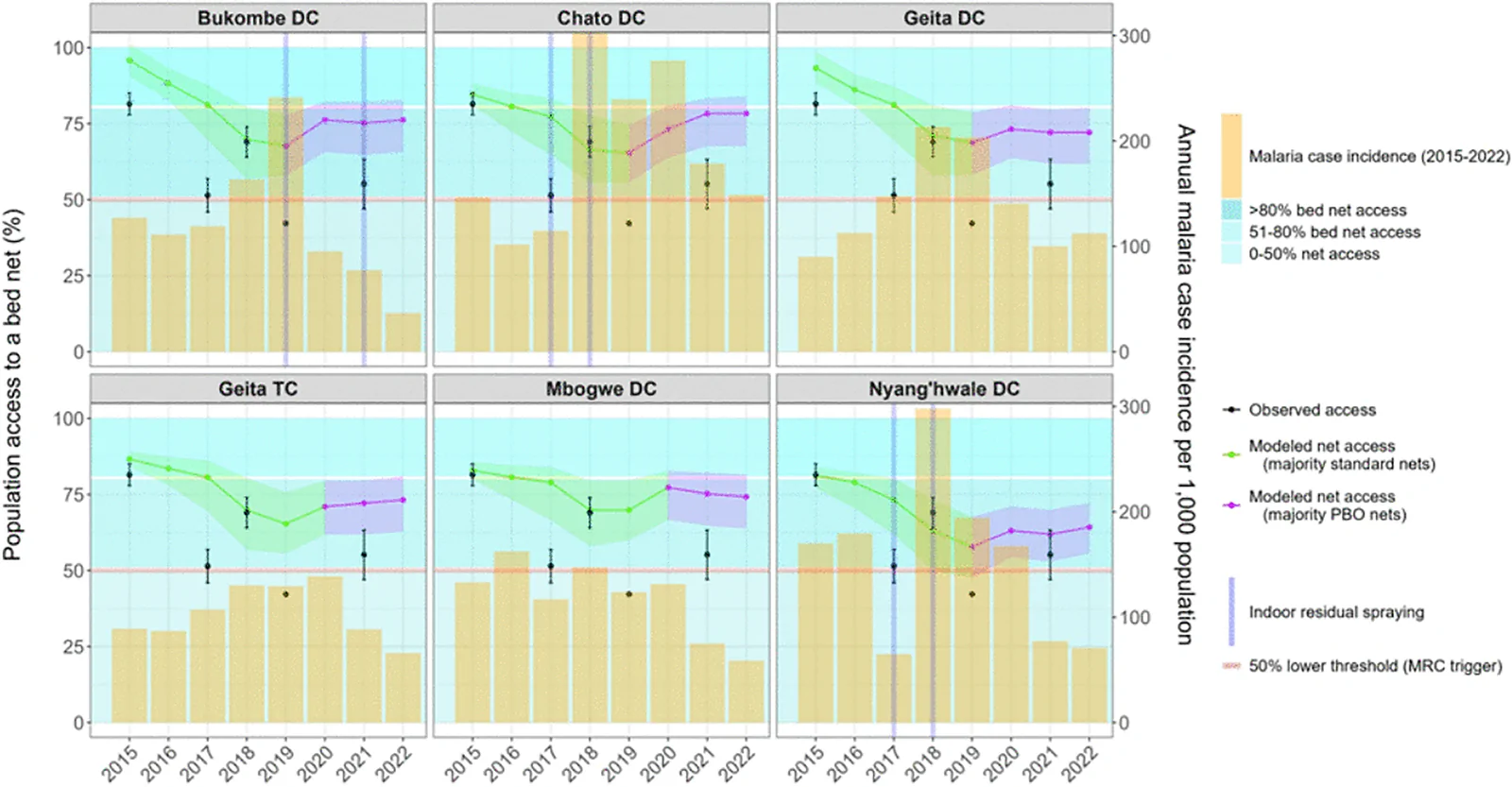
Population access to a bed net and annual malaria case incidence.

### Malaria incidence patterns

Malaria incidence rates in 2022 ranged from 0.5 cases per 1,000 population in Mbulu Town Council (Manyara Region) to 346.0 cases per 1,000 population in Kisarawe District Council (Pwani Region). Over half of the councils (100 of 184) recorded annual malaria incidence rates below 60 cases per 1,000 population, thereby meeting the NMCP 2025 target. This value increased from about 33% in 2021. Just over one quarter (51 of 184) of councils recorded between 60 and 120 cases per 1,000 population, and about 18% (33 of 184) had more than 120 cases per 1,000 population. More than 70% of councils showed a declining malaria incidence trend since 2018, 50 of 184 had stable incidence, and only one council (Bariadi Town Council in Simiyu region) showed an increasing incidence trend.

Geita region had a high malaria case incidence (Fig 5) compared with the NMCP target of fewer than 60 new cases per 1,000 population, except in two councils (Bukombe and Mbogwe). Chato had the highest malaria incidence (149 cases per 1,000 population) as of 2022, whereas Bukombe had the lowest rate (37 cases per 1,000 population). In all but one district (Chato), the monthly malaria case incidence rates in Fig 6 showed a downward trend over time (from January 2018 to December 2022). In addition, we used the slope of the best-fit line from a simple linear regression model to determine whether the time-series data showed an increasing, decreasing, or stable trend. Trends were considered statistically significant when the p-value for time was ≤ 0.05; otherwise, the slope was not considered significantly different from zero. In Chato district (Fig 7), malaria incidence increased until 2020, then declined sharply in 2021, possibly due to reduced outdoor activity during the COVID-19 pandemic. Year-over-year changes in annual malaria case incidence have fluctuated with increases/decreases across all councils.

**Fig 5:**
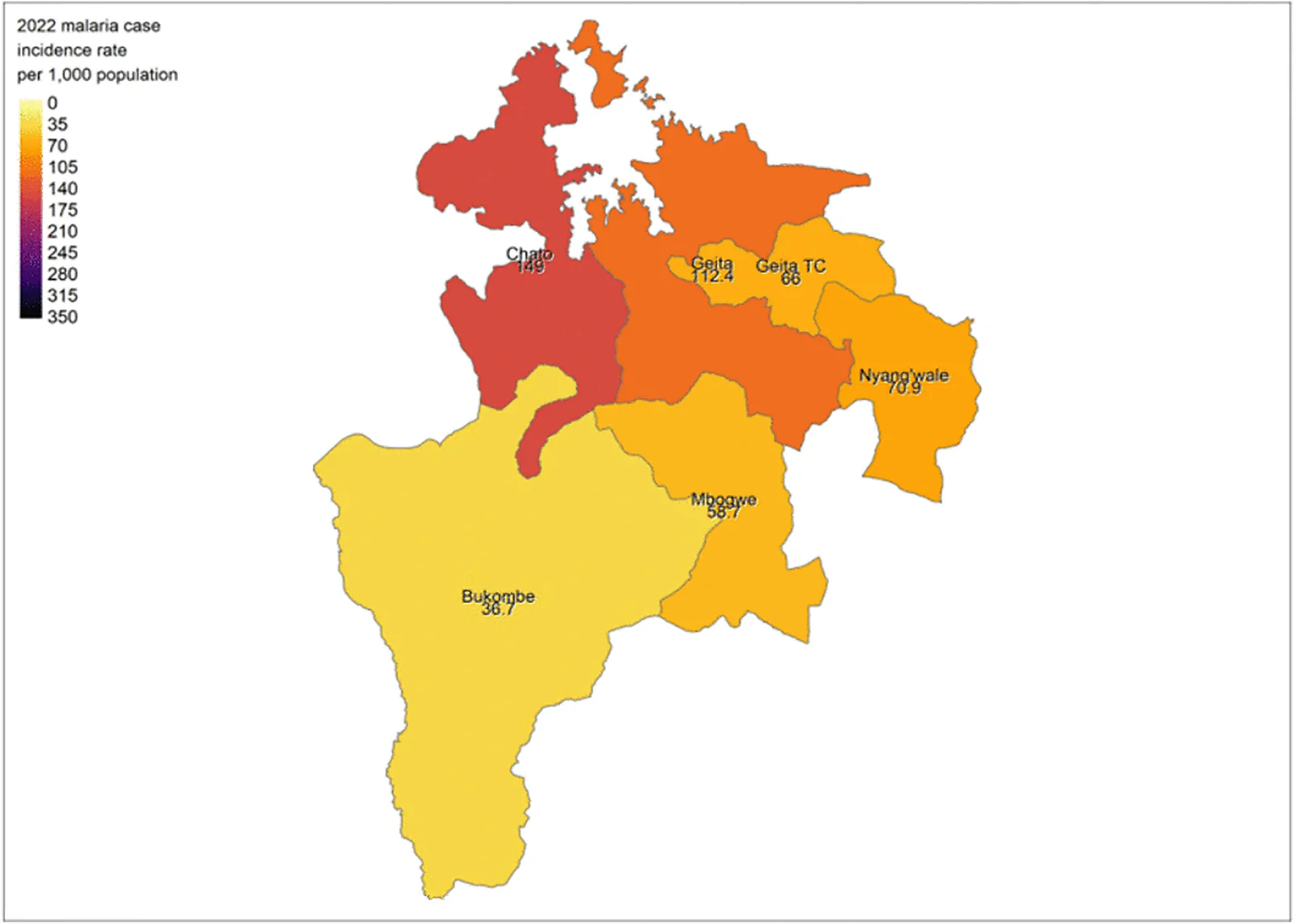
2022 Malaria case incidence rate per 1,000 population.

**Fig 6:**
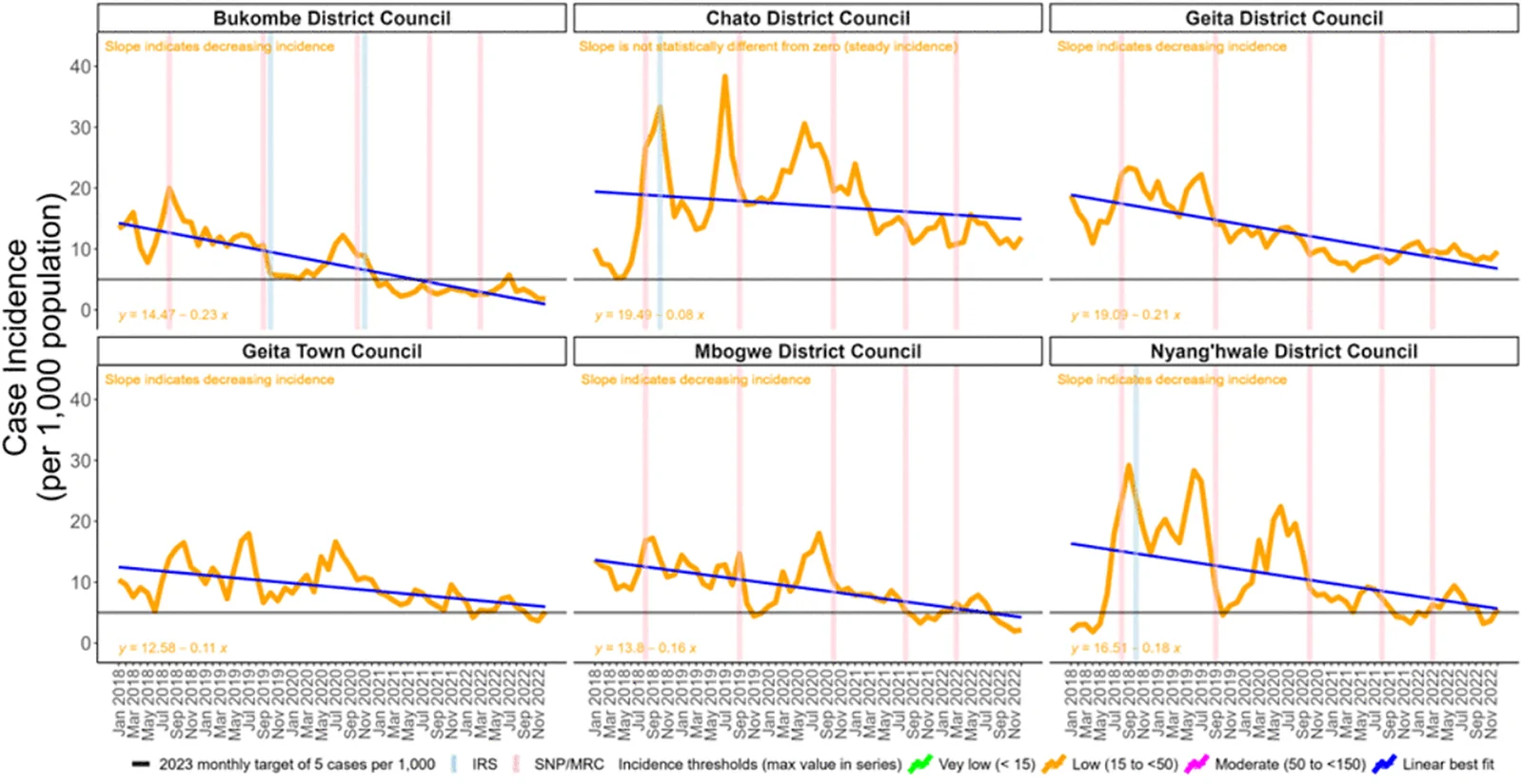
Monthly time trend of malaria case incidence – Jan 2018 to Dec 2022.

**Fig 7:**
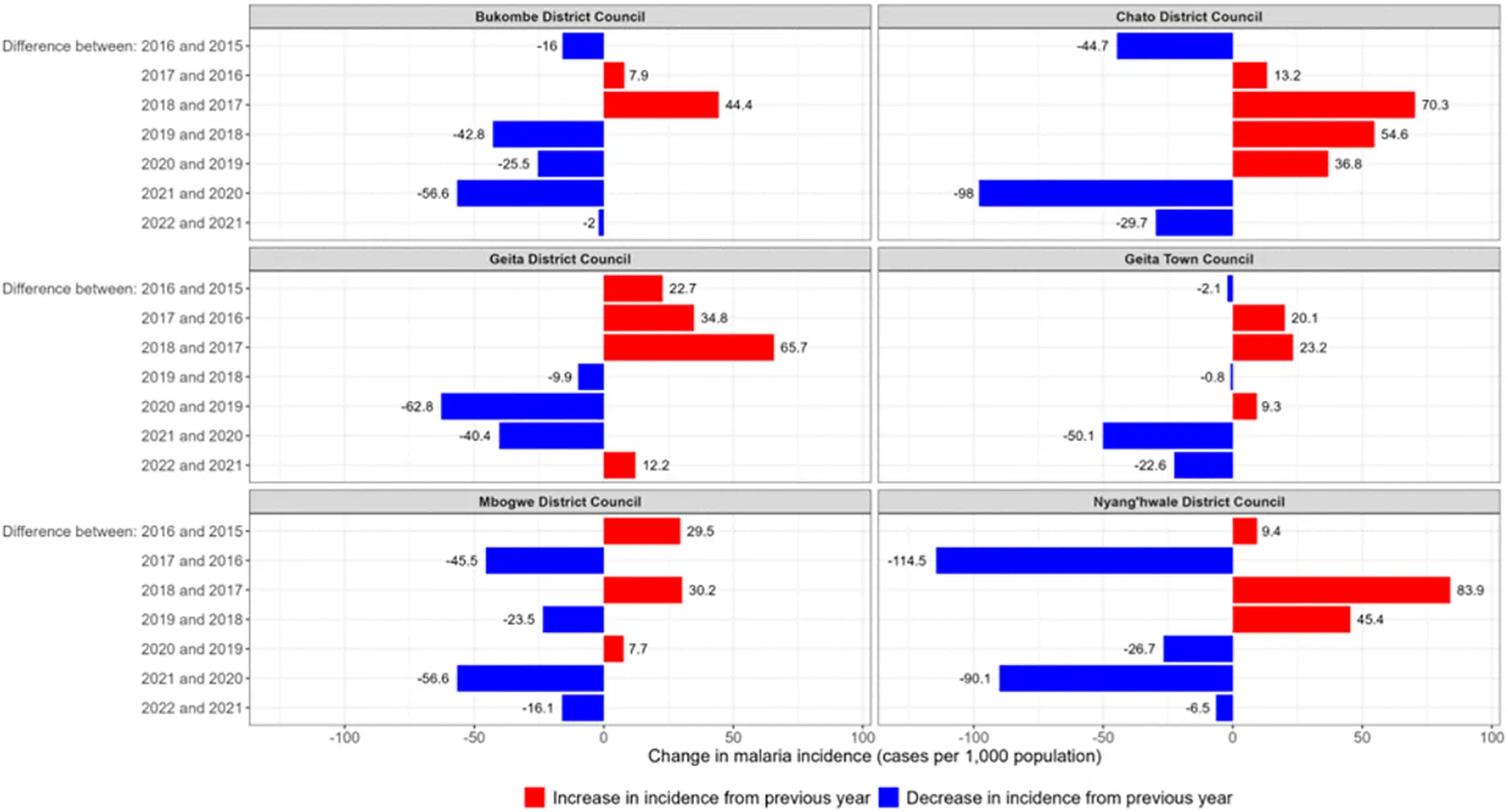
Year-over-year change in malaria case incidence rate per 1,000 population – 2015 to 2022.

### Rainfall and seasonality

Rainfall patterns were categorized as either unimodal or bimodal across regions. In rural councils, malaria transmission typically peaked following heavy rains, consistent with known seasonal dynamics. In contrast, in urban councils (town councils, municipalities, and cities), the rainy season had little to no impact on malaria cases.

For example, in the Geita region, the annual rainfall data show a bimodal rainy season across all councils, with peaks in March/April and then around November. Rainfall is low from June through August. In some councils (Chato and Nyang’hwale), malaria cases have been more prominent since the March–May rains. Other councils did not exhibit this more noticeable trend.

### Insecticide resistance

Data mining of IRS/entomological monitoring reports revealed references to insecticide resistance testing across 42 councils in mainland Tanzania since 2018 [55–57]. Among these 42 councils, 40 (95.2%) showed evidence of pyrethroid resistance, and two (4.8%) councils showed no evidence of resistance to any chemicals tested in 2020/2021 (Kilombero DC and Mtwara MC).

For example, in Geita region, insecticide resistance tests were conducted to inform the appropriate selection of insecticides for ITNs and IRS. In all councils where insecticide resistance testing was conducted (Bukombe, Chato, Nyang’hwale, and Geita), varying levels of pyrethroid resistance to permethrin, deltamethrin, and alphacypermethrin were detected between 2018 and 2022. Except for Bukombe, all insecticides evaluated for indoor residual spraying (bendiocarb, pirimiphos-methyl, and DDT) achieved mortality rates of ≥98%, indicating that local mosquito populations remained susceptible to these chemicals (Table 1) [56,57].

**Table 1:** Insecticide resistance testing in the Geita region*.*

		Permethrin (0.75%)		Deltamethrin (0.05%)		Alphacypermethrin (0.0025%)	
Ento-surveillance period	Council	Number Exposed	Percentage (%) (95% Confidence Interval)	Number Exposed	Percentage (%) (95% Confidence Interval)	Number exposed	Percentage (%) (95% Confidence Interval)
May-2022 to June-2022	Bukombe		88.75		72.5		58.75
Oct-2020 to Sep-2021	Bukombe	80	94(55; 100)	80	96 (66; 100)	80	89 (38; 100)
Apr-2020 to Jun-2020	Chato	83	40 (16; 63)	84	56 (32; 80)		
Apr-2020 to Jun-2020	Nyang’hwale	80	21 (2; 41)	80	23 (2; 43)		
Sep-2018 to Oct-2019	Geita		53 (47; 59)		66 (56; 76)		
		**Bendiocarb (0.1%)**		**Pirimiphos-Methyl (0.25%)**		**DDT**	
Ento-surveillance period	Council	**Number Exposed**	**Percentage (%) (95% Confidence Interval)**	**Number exposed**	**Percentage (%) (95% Confidence Interval)**	**Number exposed**	**Percentage (%) (95% Confidence Interval)**
Oct-2020 to Sep-2021	Bukombe			80	100(100; 100)		
Apr-2020 to Jun-2020	Chato	83	100(100; 100)	84	100(100; 100)		
Apr-2020 to Jun-2020	Nyang’hwale	80	100(100; 100)	80	98 (90; 100)		
Sep-2018 to Oct-2019	Geita		100(100; 100)		100(100; 100)		100 (100; 100)

Yellow: Resistant (≤90%), Blue: Resistant to be confirmed (90%–97%), Green: Susceptible (≥98%).

### Vector species composition

Entomological surveillance confirmed a shift in vector-species dominance. *Anopheles arabiensis*, known for its outdoor feeding behavior, has become the predominant vector in many areas, followed by *Anopheles funestus* and *Anopheles gambiae s.s*. Councils with a high presence of *Anopheles funestus* reported malaria incidence rates exceeding 60 cases per 1,000 population, despite high ITN coverage.

For example, in Geita region, *Anopheles arabiensis* was the most common primary vector based on recent entomological surveillance in three councils (Chato, Geita, and Nyang’wale), whereas *Anopheles funestus* was common in Geita DC. The distribution of other vectors is shown in Table 2 below. Traditionally, *Anopheles arabiensis* contributes to outdoor residual malaria transmission, as indoor interventions (ITNs and IRS) do not significantly affect the vector. Examples of districts with high proportions of *Anopheles arabiensis* and high annual malaria incidence rates include Chato DC and Geita DC in the Geita region, Kakonko DC and Kigoma DC in the Kigoma region, and Misungwi DC in the Mwanza region. Consequently, outdoor interventions such as larviciding may be needed to interrupt and further reduce malaria transmission.

**Table 2:** Summary matrix table of malaria vector control interventions and key data elements.

	Vector control coverage				Malaria incidence		Malaria prevalence	Entomological indicators								Wealth proxies	
	% ITN access (2021)	% ITN use (2021)	% pregnant women that received an ITN at ANC (2021)	% infants that received an ITN at measles booster (2021)	Annual confirmed malaria cases per 1,000 population (2021)	Annual confirmed malaria cases among pregnant women per 1,000 women attending ANC1 visits (2021)	Malaria parasite prevalence among primary school enrolled pupils (2019)	Primary malaria vector based on probability of occurrence through spatial modeling of historical data (2012-2014)			Primary malaria vector based on recent entomological surveillance (2016-2021)			Sporozoite rate	Entomological inoculation rate	Years of educational attainment for women of reproductive age (2014 - population weighted)	Prevalence of improved housing [0=low; 1=high]] (2015)
								An. arabiensis	An. funestus	An. gambiae	An. arabiensis	An. funestus	An. gambiae				
Bukombe District Council	75.23	62.44	89.02	48.79	38.74	124.24	30.0-49.9	0.36	0.29	0.47	92%	3.5	0%	0.3%	1.9	4.56	0.08
Chato District Council	78.32	65.00	92.56	54.88	178.73	238.89	30.0-49.9	0.58	0.27	0.71	100%	--	0	2.4%	17.1	4.39	0.10
Geita District Council	72.12	59.86	77.76	42.02	100.20	181.84	50.0-74.9	0.72	0.26	0.67	26%	65%	0%	2.0%	1.7	5.00	0.11
Geita Town council	72.12	59.86	92.15	51.41	88.60	104.03	30.0-49.9	0.72	0.23	0.63	80%	1%	7%	2.9%	6.5	5.99	0.16
Mbogwe District Council	75.23	62.44	84.54	49.61	74.89	162.90	50.0-74.9	0.86	0.21	0.64	N/A	N/A	N/A	--	--	4.42	0.09
Nyang’hwale District Council	61.90	51.38	91.84	47.17	77.45	182.74	50.0-74.9	0.9	0.16	0.6	99%	--	1%	0.9%	1.1	5.03	0.09

### District-level profile in relation to ITN coverage, malaria case incidence trends, and pyrethroid resistance for the Geita region

With reference to Fig 8, modeled ITN access decreased between 2015 and 2019. Despite an increase in 2020 due to an influx of nets via SNP, net access levels generally leveled off from 2020. Monthly malaria case incidence rates typically decline over time, except in the Chato council, which shows a long-term stable trend. ITN access is in the moderate range (51–80% access) for the entire region based on modeled estimates. We combined ITN population access estimates and confirmed malaria case trend data (2018–2022) to classify each district into one of the nine categories shown in the Fig 8 legend. We also considered pyrethroid resistance where detected.

**Fig 8:**
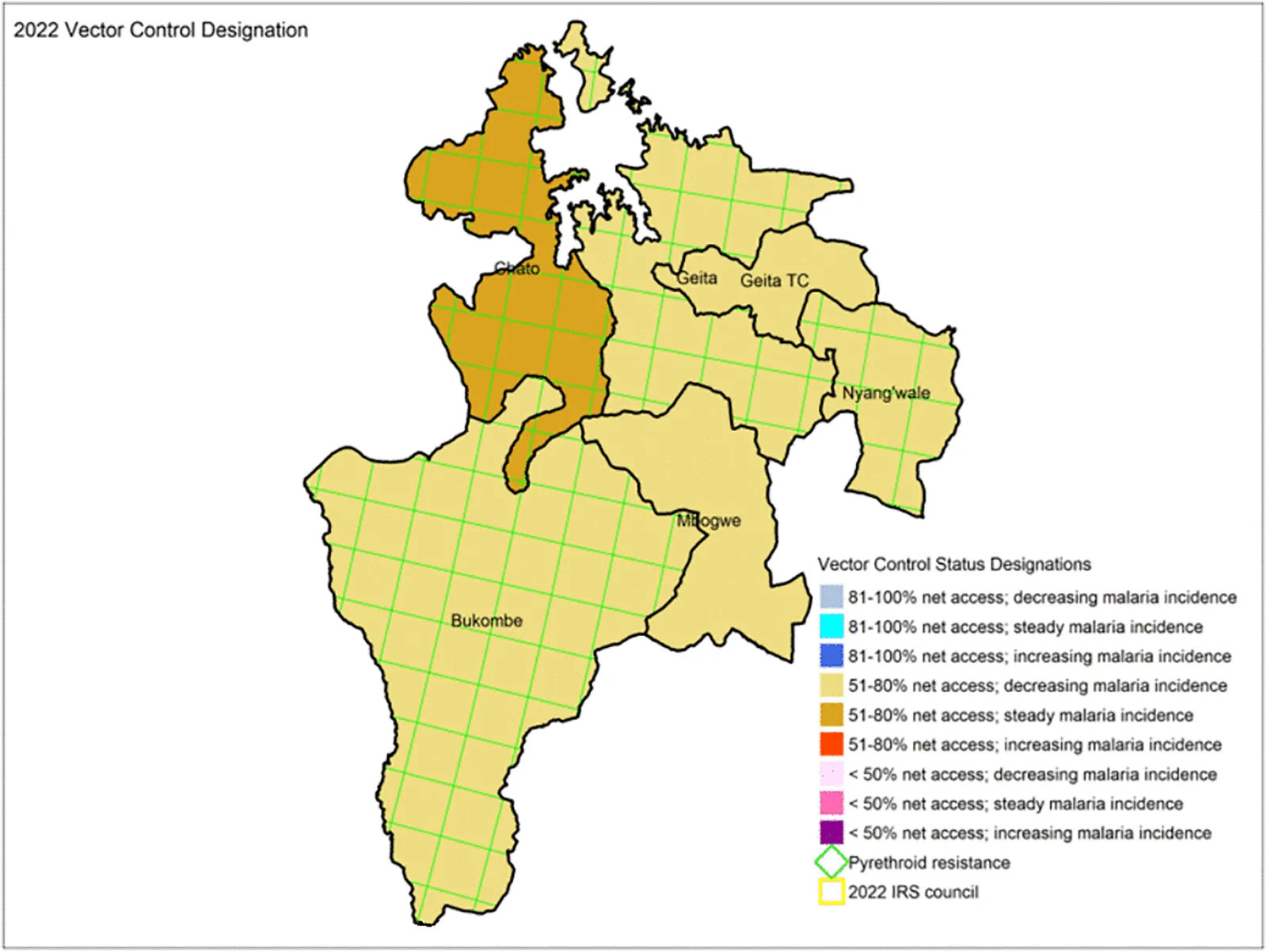
2022 vector control status designations.

### Classification summary

Half of the councils (92) had declining monthly malaria incidence and at least 50% ITN population access, with no evidence of insecticide resistance. Even among the 40 mainland councils with evidence of pyrethroid resistance, vector control coverage was above 50%. In one Council, Kinondoni Municipal in Dar es Salaam, ITN access was only 33.3%; malaria case incidence, by contrast, was either declining or holding steady across all 40 councils. Fig 9 depicts all mainland councils by vector-control designations.

**Fig 9:**
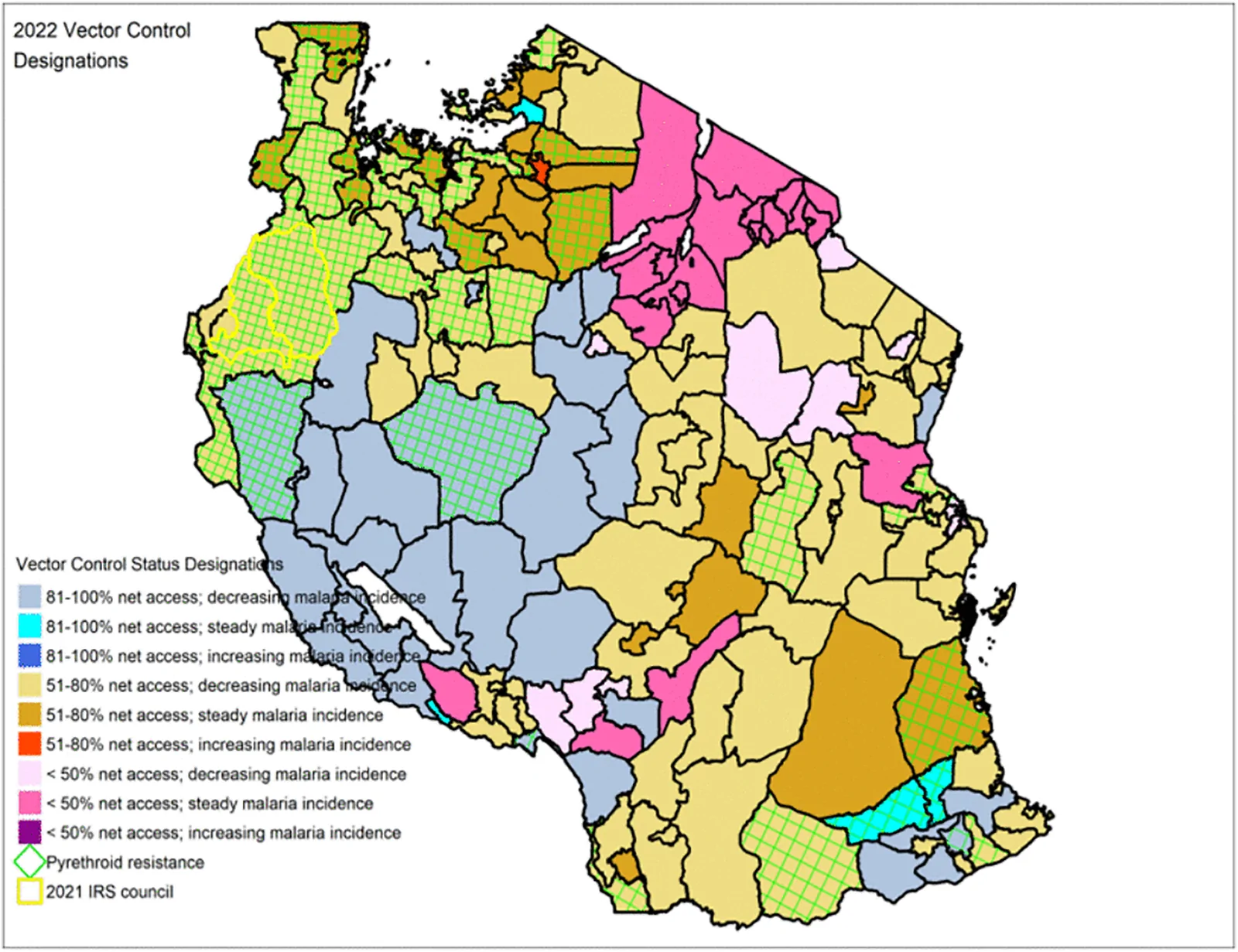
2022 vector control designations.

ITN population access estimates and confirmed malaria case trend data (2018–2022) were combined to classify each district into one of the nine categories depicted in the figure legend. The added context of pyrethroid resistance was accounted for where detected.

Only 18.5% of councils (34 of 184) achieved ITN access levels of 81–100%, yet none demonstrated an increasing trend in monthly malaria incidence. More than half of the councils (105 of 184; 57.1%) fell within the 51–80% ITN access category and exhibited declining malaria incidence over time. Table 3 provides the complete distribution of councils across vector control designations.

**Table 3:** Cross-tabulation of vector control status designations.

	Population Access to a Bed Net			
Monthly Malaria Case Incidence Trend	<50% net access	50% to ≤80% net access	81% to 100% net access	Total
**Decreasing malaria incidence**	12	83	38	133
**Steady malaria incidence**	20	25	5	30
**Increasing malaria incidence**	0	1	0	1
**Total**	**32**	**109**	**43**	**184**

## Discussion

### To depict bed net distribution channels and net crop, show IRS and MRC time points, and describe district-level population access to bed nets

The AVCR was developed using ITN distribution data compiled across various delivery channels since 2004. These included periodic mass distribution approaches, such as UCCs and MRCs, as well as the “Keep Up” strategy, through which ITNs were continuously distributed to infants and pregnant women at reproductive and child health (RCH) clinics and to primary school pupils through the School Net Programme (SNP) [2,48]. Given these multiple distribution channels, regular monitoring of population access to ITNs was essential to determine whether coverage remained above target thresholds and to inform annual ITN quantification and distribution planning. However, council-level data on ITN access were largely unavailable. Most household surveys provide estimates only at the regional level, except the Mobile Survey, which generated council-level estimates of ITN access [51]. The AVCR therefore fills an important information gap by providing council-level estimates that can support targeted planning and help sustain optimal ITN coverage for malaria prevention.

The modeled population access to ITNs estimates correlated with observed estimates derived from general population-based surveys in most regions. For example, the modeling exercise results showed that the average council-level measure of population access to an ITN was 63.9% in 2021, while the average regional-level measure of this same indicator was 57.2%, as calculated from the early 2021 mobile phone survey [25,51].

To depict annual district-level malaria case incidence, monthly malaria case incidence trends, year-over-year differences in annual malaria case incidence, and IRS and MRC time points. The study established trends in vector control interventions and malaria transmission in Tanzania (both monthly and annual malaria case incidence) [45]. In general, modeled ITN access increased from 2015 to 2017, then gradually decreased in 2021 [58]. However, in SNP councils, the modeled access was maintained for several years [58]. ITN distribution through mass campaigns results in high net access, which declines over time unless sustained by other continuous bed net distribution channels, namely, SNP, ANC, and EPI. Therefore, the community should adopt other continuous net distribution mechanisms to maintain optimal net access [49].

Monthly malaria case incidence trends were either decreasing in most councils or holding steady in a few areas, including Chato. However, malaria case incidence increased in three councils: Musoma, Kilombero, and Kilwa. Year-over-year changes in annual malaria case incidence have fluctuated. Multiple factors can explain these trends in malaria transmission, including access to and use of ITNs, entomological profiles (species distribution and insecticide resistance), and human and environmental conditions (elevation, slope, temperature, and precipitation) [12,17].

### To depict annual rainfall and malaria case incidence patterns

This study shows that all regions in Tanzania fall into two categories: unimodal or bimodal rainfall patterns. These rainfall patterns have been observed in other African countries and have been shown to influence malaria transmission [17]. Rainfall patterns were associated with malaria transmission across all rural areas, with transmission peaking two weeks after heavy rains. However, in municipal and city council areas, the rainy season had little to no impact on malaria case incidence. This may be attributable to housing improvements (e.g., closed eaves, indoor wire-mesh installation, and window installation), socioeconomic status, urban agriculture, and improved access to appropriate diagnosis and treatment in Dar es Salaam city [59]. In Uganda, higher urbanicity was associated with lower mosquito densities, reduced malaria transmission, and lower prevalence. These trends were similar to previous findings elsewhere [60]. Other factors, apart from weather, that contribute to increases in malaria incidence rates should be investigated, particularly in urban areas with complex ecosystems. Highland councils, despite peaks in bimodal rainfall, have very low malaria incidence rates because lower temperatures reduce vector reproduction and survival.

Adaptive strategies for malaria control should be emphasized, including using climate data to inform early-warning systems for epidemic preparedness and response. This could include timing malaria and vector control interventions—i.e., IRS, distribution and use of ITNs, and encouraging early diagnosis and treatment—alongside efforts to reduce mosquito breeding sites. Community engagement, education, and proactive efforts to address climate-related challenges can enhance malaria control and protect the health and well-being of vulnerable populations. Tailoring messages to be culturally and contextually relevant is effective. Multi-sectoral climate change adaptation strategies can strengthen the capacity and resilience of health systems and communities, especially in regions with predicted climatic suitability for future emergence and re-emergence of malaria [5,14–16,18–22].

In this review, we found that malaria vectors were fully susceptible to clothianidin and partly susceptible to pirimiphos-methyl in all monitored councils except Igunga and Kakonko. Nonetheless, it remains essential to continue monitoring the effectiveness of these insecticides in IRS and manage resistance proactively. Maintaining the effectiveness of clothianidin and pirimiphos-methyl involves rotating insecticides used in IRS, as stipulated by the National Insecticide Resistance Management Plan [61]. The observed resistance to permethrin, deltamethrin, and alphacypermethrin is attributed to insecticide pressure from the cumulative effect of long-term ITN use (i.e., nets treated with permethrin or deltamethrin), with very few treated with alphacypermethrin. Other factors that might have contributed to the observed pyrethroid resistance include the extensive use of pyrethroids in agriculture and IRS, which used lambda-cyhalothrin (a pyrethroid) for spraying in the Lake zones.

High resistance levels were widely distributed and could lead to programmatic failure of insecticide-based vector control tools, including ITNs. However, well-maintained ITNs can still provide a physical barrier and prevent bites, regardless of their insecticide activity. When pyrethroids no longer kill resistant vectors, they can still act as repellents and/or irritants. These effects could interfere with disease transmission even when an insecticide kills no mosquitoes [62].

### To describe the district vector composition in relation to the occurrence of malaria incidence, given vector control interventions in place

The primary malaria vectors in most areas were members of the *Anopheles gambiae* complex and the *Anopheles funestus* group. Following the scaling up of indoor vector control interventions (ITNs and IRS), the population of *Anopheles gambiae s.s*. has decreased dramatically, while *Anopheles arabiensis* has become the predominant malaria vector in various areas of mainland Tanzania [36,63,64].

Similarly, findings show that the incidence of Anopheles gambiae s.s. steadily decreased in most council areas, while the incidence of Anopheles arabiensis and Anopheles funestus gradually increased. Similarly, areas with high *Anopheles funestus* occurrence exhibited steady annual malaria incidence rates exceeding the national target of 60 cases per 1,000 population. This is due to their high vectorial capacity for malaria transmission*,* coupled with high resistance to almost all insecticide classes, particularly pyrethroids [65]. Traditionally, *Anopheles arabiensis* contributes to outdoor residual malaria transmission, as it is not significantly affected by indoor interventions (ITNs and IRS). Consequently, outdoor interventions such as LSM, particularly larviciding, may be needed to reduce outdoor malaria transmission [27].

Deployment of new-generation nets such as Interceptor G2 and PBO nets, and/or IRS is highly recommended in councils where *Anopheles funestus* was the dominant malaria vector, and annual malaria incidence rates exceeded the NMCP’s target, as in Nachingwea DC (114.26 cases per 1,000 population) and Liwale DC (402.3 cases per 1,000 population) [66].

### To establish an overall district-level profile of ITN coverage, malaria case incidence trends, and pyrethroid resistance

Survey data on ITN access at the council level were unavailable for this study [25]. Nevertheless, the study’s modeled estimates of ITN access indicated that most councils fall within the moderate range (51–80%). Moreover, in the councils with 81–100% ITN access, incidence rates were comparatively low. This suggests that maximizing ITN access reduces the malaria burden, as seen in southern regions, where malaria case incidence coincides with low ITN access, insecticide resistance, and poor housing. The scale-up of ITN ownership has coincided with the gradual spread of pyrethroid resistance nationwide [45,57].

Based on synergy assays with PBO, most test areas exhibited a broad distribution of oxidase-based metabolic resistance. This implies that standard ITNs may be less effective in protecting individuals from malaria transmission. Unfortunately, only 9 regions (34%) were distributing PBO nets at the time; therefore, PBO nets needed to be deployed in the remaining areas, especially those with moderate-to-high malaria transmission. Other resistance mechanisms observed in the country, including nonspecific esterases, may contribute to metabolic resistance, as reported in previous studies [55].

Despite limited IRS coverage due to funding constraints, only four regions—15% of all areas with high malaria intensity in the Western and Lake zones—had coverage. This significantly reduced malaria incidence, underscoring the need to further consider the role of IRS, except in Chato Council, where it has not reduced malaria case incidence. Consequently, IRS and PBO nets could be further implemented in southern areas (Lindi, Mtwara, and Ruvuma) where malaria transmission is as high. Historically, IRS or ITNs have been proven effective tools for reducing receptivity and preventing epidemics and the resurgence of malaria, and could feasibly be introduced, even in the context of a complex emergency [3,46,67–69].

The councils where both PBO nets and IRS were deployed include Chato and, to some extent, Nyang’wale, although malaria incidence rates remained high in those areas, possibly due to the predominance of *Anopheles arabiensis*. Therefore, ITNs and IRS may not be sufficient to reduce malaria incidence in such areas. Larviciding as a supplementary approach seems promising, as it can control mosquitoes during their immature stages (in breeding habitats) where they cannot escape. Exhaustive coverage of larviciding at a few fixed, readily identifiable mosquito breeding sites could overcome pyrethroid resistance, since larviciding is a crucial tool for malaria control and elimination [27,70,71].

### Limitations and considerations

Some notable limitations arose from council-level ITN access estimates, including changes in council boundaries/designations over time. This meant that councils established before the 2012 census had missing population data, necessitating the reallocation of historical ITN distribution records. The estimates left several variables unaccounted for: the 2012 census projections did not capture population shifts and within-subnational migration; the acquisition of ITNs through the private commercial sector; variability in net median lifespan across the country; and oversaturation of ITNs in some households. The SNP targets households with children attending primary school (around 50% of households in Tanzania) and encourages families to redistribute ITNs within households; however, some do not.

Despite these limitations, the present approach remains a promising method for estimating ITN access, particularly at the council level, where regular observational surveys were too expensive, time-consuming, or logistically impossible to implement. It is a given that the NMCP strategic plan (2021–2025) and the current strategic plan 2026–2030 [2,72] interventions need to be stratified and targeted to the council level. Modeling ITN access using country-specific net decay rates, council-level population data, and ITN distribution data was a promising approach for monitoring ITN coverage at the subnational level and across household surveys in Tanzania and other countries. This case study approach was helpful in Tanzania’s context and beyond, as ITN access estimates were needed to inform proper quantification during annual planning.

More innovative work has digitized this report/review as a tool for assessing ITN access and analyzing vector control intervention data, generating visualizations, and making findings easily accessible on the DHIS2 platform. Eventually, we hope to be integrated into routine decision-making and guide the effectiveness of vector control interventions at various administrative levels in Tanzania.

## Conclusion

Vector control tools help us understand local vector behavior, which determines disease endemicity [73]. Therefore, deploying ITN, IRS, and LSM helps control malaria [58,72–75]. Modeling population ITN access using net crop is a mainstream method for calculating subnational ITN coverage and can inform quantification at the council level.

The findings indicate that in all councils across all regions where *Anopheles arabiensis* and *Anopheles funestus* were predominant, annual malaria incidence rates exceeded the NMCP’s target of fewer than 60 new cases per 1,000 population, regardless of high ITN and/or IRS coverage. Therefore, the integrated vector management approach should be emphasized, with a special focus on integrating larval source management and larviciding into existing effective vector control interventions to control the observed outdoor residual malaria transmission and accelerate malaria elimination efforts in the country.

Moreover, in rural areas, transmission peaks occur just after heavy rains, whereas in municipal and city district councils, the rainy season does not affect malaria cases. Therefore, additional factors contributing to malaria incidence in urban settings should be investigated, e.g., by strengthening entomological surveillance to assess the potential invasion of *Anopheles stephensi.*

High resistance across the country could lead to programmatic failure of ITNs if no alternative ITNs are deployed. Therefore, the appropriate targeted deployment of WHO-approved new-generation nets, i.e., Dual AI and IRS with effective insecticides, is recommended to manage resistance.

Given the impact of climate change on malaria transmission, an adaptive approach to malaria control should be emphasized, including the timing of deploying vector control interventions (e.g., IRS), the distribution and use of ITNs to maximize impact, and the use of climate data to inform early warning systems for epidemic preparedness and response.

### Recommendation

NMCP and other malaria programs should use the vector control council profiles to prioritize interventions in areas with the highest malaria burden, allocate resources more effectively, and adjust strategies based on the latest district-level data. Thus, council-level profiles enable more precise, informed decisions, leading to more cost-effective and impactful malaria control efforts.

Strengthen quantification processes and use the population-quantifier approach, especially for ITNs, to ensure sufficient ITNs are procured and distributed through appropriate channels to maintain the recommended net access level (at least 80%).

Consider deploying WHO-approved new-generation ITNs in as many regions as possible to manage pyrethroid resistance and maintain reductions in annual malaria incidence. Expand insecticide resistance monitoring to assess the effectiveness of ongoing vector control interventions, and conduct malaria vector bionomic studies to establish vector profiles.

Given the complexity of the vector profile, it is further recommended that the integrated vector management approach be implemented, with a special focus on integrating LSM into existing effective vector control interventions to control the observed outdoor residual malaria transmission, in which *Anopheles arabiensis* was dominant.

For subnational data segregation and analysis, consider changes in council boundaries/designations over time, as well as population shifts, to ensure the representativeness of new councils and migrated/shifted populations in particular subnational areas.

Anthropological studies should explore human behavior in relation to rising annual malaria incidence rates, despite vector control interventions implemented in certain district councils (e.g., Bariadi TC in the Simiyu region).

The climate change agenda should be explicitly incorporated in the next National Malaria Strategic Plan, particularly on the timing of deploying vector control interventions (e.g., IRS) and the distribution and use of ITNs to maximize impact.

## Supporting information

S1 Fig Tanzania map shows the macro-stratification of the subnational (councils) level by malaria burden. According to the Supplementary Malaria Midterm Strategic Plan 2018–2020 National Malaria Control Programme (NMCP) stratified Tanzania Mainland into four epidemiological strata (very low, low, moderate, and high malaria transmission) and one operational stratum (urban councils), with councils set as the operational level main transmission classes: (1) malaria-free, unstable transmission, and less than 1% parasite rate; (2) malaria low transmission (1% to less than 10% parasite rate) (3) moderate transmission (10% to less than 30% prevalence rate); and (4) malaria high (>30% parasite rate) transmission.
(DOCX)

## Abbreviations

ANC: Antenatal clinic
AVCR: Annual Vector Control Report
EPI: Expanded programme for Immunization
IRS: Indoor Residual Spraying
ITN: Insecticide-treated net
LSM: Larval Source Management
MRC: Mass Replacement Campaign
RCH: Reproductive and Child Health Services
SNP: School Net Programme
WHO: World Health Organization
Dual AI: Dual active-ingredient insecticide-treated nets
PBOs: Piperonly butoxide insecticide-treated nets
SES: Socioeconomic-status
Interceptor G2: is a next generation insecticide-treated nets and G2 stands for Second Generation/upgrated ITN
TNVS: Tanzania National Voucher Scheme
