# Supplementary material for: Strategies and approaches to vector control effectiveness toward the elimination of malaria in mainland Tanzania: An implementation case study analysis
Source: PLOS Glob Public Health. 2026 Oct 22;6(10):e0005574. doi: 10.1371/journal.pgph.0005574 (PMC13690203; doi:10.1371/journal.pgph.0005574)
Supplement: Supplementary file 1 [file pgph.0005574.s003.pdf]

3rd December 2025

Re: PLOS Global Public Health Decision: Revision required [PGPH-D-25-02234] - [EMID:e965ae32ee5da6db]

PGPH-D-25-02234
Strategies and approaches to vector control effectiveness towards malaria elimination in mainland Tanzania: An implementation case study analysis
PLOS Global Public Health

Dear
Dr. Kate Anne Manalo Dimayuga
Editor
PLOS Global Public Health

Thank you for taking the time to consider our manuscript for publication in the PLOS Global Public Health Journal and for providing your valuable comments.

We appreciate the input you have provided, which will help improve this manuscript. We have modified the paper in response to the extensive and insightful editor and reviewers' comments. We have added additional explanatory items that the editor identified. Furthermore, we have revised certain sections of the manuscript and hope this addresses the referee's remarks. We have responded to the comments point by point in the table below.

Journal requirements

	**Comments**	**Responses**
1	Please remove the old manuscript file and retain only the revised files.	a. Thank you. We have adhered to the Journal's requirements by removing the old file and retaining only the revised files.
2	We do not publish any copyright or trademark symbols that usually accompany proprietary names, eg (R), (C), or TM (e.g. next to drug or reagent names). Please remove all instances of trademark/copyright symbols throughout the text, including ® on page 20, 21.	Thank you for your time and valuable comments. We have removed all the trademarks/copyright symbols throughout the text in the revised manuscript.

We look forward to hearing from you regarding our submission. We would be happy to respond to any further questions or comments you may have. Thank you once again for your most valuable time and input.

Kind regards

Mponeja Gitanya, BSc, MPH

Corresponding author

Principal Investigator: **Strategies and approaches to vector control effectiveness towards malaria elimination in mainland Tanzania: An implementation case study analysis**

KCMC University, Moshi, Kilimanjaro, Tanzania

P.O.BOX 2240, Moshi,

Kilimanjaro, Tanzania

Mob: +55 713 719909
