# Supplementary material for: Strategies and approaches to vector control effectiveness toward the elimination of malaria in mainland Tanzania: An implementation case study analysis
Source: PLOS Glob Public Health. 2026 Oct 22;6(10):e0005574. doi: 10.1371/journal.pgph.0005574 (PMC13690203; doi:10.1371/journal.pgph.0005574)
Supplement: Supplementary file 2 [file pgph.0005574.s001.docx]

**S1 Fig. Tanzania map shows the macro-stratification of the subnational (councils) level by malaria burden**

Source: Supplementary Malaria Midterm Strategic Plan 2018-2020.

*Base map source: Tanzania administrative boundaries. Humanitarian Data Exchange (HDX), dataset provided by the Tanzania National Bureau of Statistics and UN OCHA ROSA, and licensed under the Creative Commons Attribution for Intergovernmental Organizations (CC BY-IGO).*
