# Supplementary material for: Strategies and approaches to vector control effectiveness toward the elimination of malaria in mainland Tanzania: An implementation case study analysis
Source: PLOS Glob Public Health. 2026 Oct 22;6(10):e0005574. doi: 10.1371/journal.pgph.0005574 (PMC13690203; doi:10.1371/journal.pgph.0005574)
Supplement: Supplementary file 3 [file pgph.0005574.s004.docx]

17^th^ March 2026

**Re: PLOS Global Public Health Decision: Revision required [PGPH-D-25-02234] - [EMID:e965ae32ee5da6db]**

**PGPH-D-25-02234R1
Strategies and approaches to vector control effectiveness towards malaria elimination in mainland Tanzania: An implementation case study analysis
PLOS Global Public Health**

Dear

Miquel Vall-llosera Camps
Staff Editor
PLOS Global Public Health

Thank you for taking the time to consider our manuscript for publication in the PLOS Global Public Health Journal and for providing your valuable comments.

We appreciate the input you have provided, which will help improve this manuscript. We have modified the paper in response to the extensive and insightful editor and reviewers’ comments. We have added additional explanatory items that the editor identified. Furthermore, we have revised certain sections of the manuscript and hope this addresses the referee’s remarks. We have responded to the comments point by point in the table below.

**Journal requirements**

|  | **Comments** | **Responses** |
| --- | --- | --- |
| 1 | If the reviewer comments include a recommendation to cite specific previously published works, please review and evaluate these publications to determine whether they are relevant and should be cited. There is no requirement to cite these works unless the editor has indicated otherwise. | Thank you for your time and valuable comments. The reviewer had no comments or recommendations on the cited, previously published work. |
| 2 | Please review your reference list to ensure that it is complete and correct. If you have cited papers that have been retracted, please include the rationale for doing so in the manuscript text, or remove these references and replace them with relevant current references. Any changes to the reference list should be mentioned in the rebuttal letter that accompanies your revised manuscript. If you need to cite a retracted article, indicate the article’s retracted status in the References list and also include a citation and full reference for the retraction notice. | Thank you for your time and valuable comments. We have reviewed and evaluated in accordance with the journal's requirements. |

**REVIEWER’S AND EDITOR'S COMMENTS**

|  | **Comments** | **Responses** |
| --- | --- | --- |
| 1 | If the authors have adequately addressed your comments raised in a previous round of review and you feel that this manuscript is now acceptable for publication, you may indicate that here to bypass the “Comments to the Author” section, enter your conflict of interest statement in the “Confidential to Editor” section, and submit your "Accept" recommendation.  Reviewer #3: All comments have been addressed | Thank you for your time and valuable comments. |
| 2 | Does this manuscript meet PLOS Global Public Health’s [publication criteria](https://track.editorialmanager.com/CL0/https:%2F%2Fjournals.plos.org%2Fglobalpublichealth%2Fs%2Fcriteria-for-publication/1/010f019c69713bb1-ff69ee6d-80a2-4dee-9513-d48c1483726d-000000/YP3FwP1flz1F0k3j4b-XeVULkGH_EwlP8uHLPhqMLvM=250)? Is the manuscript technically sound, and do the data support the conclusions? The manuscript must describe methodologically and ethically rigorous research with conclusions that are appropriately drawn based on the data presented.  Reviewer #3: Yes | Thank you for your time and valuable comments. |
| 3 | Has the statistical analysis been performed appropriately and rigorously?  Reviewer #3: Yes | Thank you for your time and valuable comments |
| 4 | Have the authors made all data underlying the findings in their manuscript fully available (please refer to the Data Availability Statement at the start of the manuscript PDF file)?  The [PLOS Data policy](https://track.editorialmanager.com/CL0/https:%2F%2Fjournals.plos.org%2Fglobalpublichealth%2Fs%2Fdata-availability/1/010f019c69713bb1-ff69ee6d-80a2-4dee-9513-d48c1483726d-000000/iiITipqGA2ylM51ehO4y8R2_7RXpcfiZoWziHaaeWQ4=250) requires authors to make all data underlying the findings described in their manuscript fully available without restriction, with rare exceptions. The data should be provided as part of the manuscript or its supporting information, or deposited to a public repository. For example, in addition to summary statistics, the data points behind means, medians and variance measures should be available. If there are restrictions on publicly sharing data—e.g. participant privacy or use of data from a third party—those must be specified.  Reviewer #3: No | Thank you for your time and valuable comments. The household-level survey data used in this analysis are publicly available from the DHS (https://dhsprogram.com/) and MICS (https://mics.unicef.org/) websites.  Moreover, since we have used secondary data (i.e., DHIS2), the other data reports will be available from the corresponding author upon reasonable request at [mpgitanya2006@yahoo.com](mailto:mpgitanya2006@yahoo.com). |
| 5 | 5. Is the manuscript presented in an intelligible fashion and written in standard English?  PLOS Global Public Health does not copyedit accepted manuscripts, so the language in submitted articles must be clear, correct, and unambiguous. Any typographical or grammatical errors should be corrected at revision, so please note any specific errors here.  Reviewer #3: Yes | Thank you for your time and valuable comments |
| 6 | Please use the space provided to explain your answers to the questions above. You may also include additional comments for the author, including concerns about dual publication, research ethics, or publication ethics. (Please upload your review as an attachment if it exceeds 20,000 characters)  Reviewer #3: Overall, this is a very useful case report with methods sure to have future impact. Thank you for compiling this.  Figure 1: Number of nets hard to relate to coverage without size of population. Similarly to the black line in figure 4, consider adding a line which is the number of nets needed each year to reach 80% coverage given median net life of 2.15 years.  To me a logical order for the methods section would be to put the malaria case incidence first. A proposed order would be: Malaria case incidence analysis, Insecticide-treated net data sources, Entomological surveillance and insecticide resistance, Rainfall and Seasonality and Council classification framework.  Figure 2: There is no mention of how PBO nets are quantified in the methods. The last methods section reads “PBO nets were advised in councils with pyrethroid resistance, provided budget constraints didn’t compromise overall ITN coverage”. However, Figure 2 has quantified this. Please explain this in the methods.  Figure 5: It is unclear from text or figure if linear regressions showed a significant trend.  Results – Vector species composition. “Other vector distributions are shown below” Where? Table 2? I cannot see this referenced in the text. Please change “below” to “Table 2”. Also, species composition is not mentioned in the table legend.  Finally, a few of the figure axis labels and legends and tables text are appearing blurry in the complied PDF. Please make sure the quality of these is in accordance to journal specifications.  Please make your data and estimations available as supplementary data for potential future analysis. While plots are shown, these pool the estimated net coverage into <50%, 50-80% and >80%, the actual estimated values are not reported. For example a table with the data from figure 9 with cases each year additionally would significantly increase the usefulness for potential future analysis. I hope you make resources openly available beyond DHIS 2. | Thank you for your time and valuable comments.   - The stock-and-flow model was used to estimate the net crop of surviving ITNs annually in mainland Tanzania (Koenker H. at el 2022). A Tanzania-specific median net lifespan of 2.15 years (CI: 1.88–2.43) was applied to decay each distribution crop based on the smooth-compact loss function developed by Nakul Chitnis and described in Koenker et al 2022, and Bhatt et al 2015. The total net crop at each council was divided by the projected population, sourced from 2012 National Bureau of Statistics census data, to calculate nets per capita (NPC) per year. ITN access was subsequently estimated by NPC using a nonparametric model derived from the Demographic Health Survey and Malaria Indicator Survey data. - The logical order for the methods section had been arranged as was suggested by reviewers; Malaria case incidence analysis, Insecticide-treated net data sources, Entomological surveillance and insecticide resistance, Rainfall and Seasonality and Council classification framework. - Thank you for the valuable comments. The quantification for the PBO nets had been explained in the methods section. - Thank you for the valuable comments. The text for the figure 5 has been explained clearly in the revised manuscript for review. - Thank you for the valuable comments. Results section on the vector species composition, was corrected as per reviewer’s comments. - Thank you for the valuable comments. All figures and tables text have been revised to be clearly to be read and as accordance to the journal specifications. - Thank you for your time and valuable comments. The authors have revised the manuscript and all data are readily available via open sources upon request from the corresponding author; [mpgitanya2006@yahoo.com](mailto:mpgitanya2006@yahoo.com). |
| 7 | PLOS authors have the option to publish the peer review history of their article ([what does this mean?](https://track.editorialmanager.com/CL0/https:%2F%2Fjournals.plos.org%2Fglobalpublichealth%2Fs%2Feditorial-and-peer-review-process%23loc-peer-review-history/1/010f019c69713bb1-ff69ee6d-80a2-4dee-9513-d48c1483726d-000000/NcxOpry3wCLg0FG557FYr7C1pTmc7tnnkrYpeccslYA=250)). If published, this will include your full peer review and any attached files.   **Do you want your identity to be public for this peer review?** If you choose “no”, your identity will remain anonymous but your review may still be made public.   For information about this choice, including consent withdrawal, please see our [Privacy Policy](https://track.editorialmanager.com/CL0/https:%2F%2Fwww.plos.org%2Fprivacy-policy/1/010f019c69713bb1-ff69ee6d-80a2-4dee-9513-d48c1483726d-000000/Ibx0gjbB_o4KQUNH9tMdI-yH-Jm3TBFkjetgzhKuOKU=250).  Reviewer #3: **Yes:** Emma Louise Fairbanks | Thank you for your time and valuable comments |

We look forward to hearing from you regarding our submission. We would be happy to respond to any further questions or comments you may have. Thank you once again for your most valuable time and input.

Kind regards

Mponeja Gitanya, BSc, MPH

Corresponding author

Principal Investigator: **Strategies and approaches to vector control effectiveness towards malaria elimination in mainland Tanzania: An implementation case study analysis**

KCMC University, Moshi, Kilimanjaro, Tanzania

P.O.BOX 2240, Moshi,

Kilimanjaro, Tanzania

Mob: +55 713 719909
