# Supplementary material for: Strategies and approaches to vector control effectiveness toward the elimination of malaria in mainland Tanzania: An implementation case study analysis
Source: PLOS Glob Public Health. 2026 Oct 22;6(10):e0005574. doi: 10.1371/journal.pgph.0005574 (PMC13690203; doi:10.1371/journal.pgph.0005574)
Supplement: Supplementary file 4 [file pgph.0005574.s005.docx]

2^nd^ June 2026

**Re: PLOS Global Public Health Decision: Revision required [PGPH-D-25-02234] - [EMID:e965ae32ee5da6db]**

**PGPH-D-25-02234R2
Strategies and approaches to vector control effectiveness towards malaria elimination in mainland Tanzania: An implementation case study analysis
PLOS Global Public Health**

Dear

Shaira Joy Petancio

PLOS Global Public Health

Thank you for taking the time to consider our manuscript for publication in the PLOS Global Public Health Journal and for providing your valuable comments.

We appreciate the input you have provided, which will help improve this manuscript. We have modified the paper in response to the extensive and insightful editor and reviewers’ comments. We have added additional explanatory items that the editor identified. Furthermore, we have revised certain sections of the manuscript and hope this addresses the referee’s remarks. We have responded to the comments point by point in the table below.

**Journal requirements**

|  | **Comments** | **Responses** |
| --- | --- | --- |
| 1 | We notice that the author list for your submission has changed during revision. In order to add or remove authors or update the order of the author byline after initial submission, we ask that authors complete an Authorship Change Request form. You may review our full authorship change policy and download the Authorship Change Request form here: [LINK] | Thank you for your time and valuable comments. The changes to the author list were corrected and made in accordance with the comments and recommendations. |
| 2 | 2. We would also like to require a written email confirmation from all authors confirming that they approve of the changes. You may upload a compiled copy of the written confirmations to your submission’s file inventory as the Item Type ‘Other. | Thank you for your time and valuable comments. We have attached the document confirming the approved changes from all authors, as per their author status, for your review. |

We look forward to hearing from you regarding our submission. We would be happy to respond to any further questions or comments you may have. Thank you once again for your most valuable time and input.

Kind regards

Mponeja Gitanya, BSc, MPH

Corresponding author

Principal Investigator: **Strategies and approaches to vector control effectiveness towards malaria elimination in mainland Tanzania: An implementation case study analysis**

KCMC University, Moshi, Kilimanjaro, Tanzania

P.O.BOX 2240, Moshi,

Kilimanjaro, Tanzania

Mob: +55 713 719909
