# Supplementary material for: Strategies and approaches to vector control effectiveness toward the elimination of malaria in mainland Tanzania: An implementation case study analysis
Source: PLOS Glob Public Health. 2026 Oct 22;6(10):e0005574. doi: 10.1371/journal.pgph.0005574 (PMC13690203; doi:10.1371/journal.pgph.0005574)
Supplement: Supplementary file 5 [file pgph.0005574.s006.docx]

17^th^ August 2026

**Re: PLOS Global Public Health Decision: Revision required [PGPH-D-25-02234R3] - [EMID: e53ae4abf84fec7f]**

**PGPH-D-25-02234R3
Strategies and approaches to vector control effectiveness towards malaria elimination in mainland Tanzania: An implementation case study analysis
PLOS Global Public Health**

Dear

Kanokwan Suwannarong, Ph.D

Academic Editor
PLOS Global Public Health

Thank you for taking the time to consider our manuscript for publication in the PLOS Global Public Health Journal and for providing your valuable comments.

We appreciate the input you have provided, which will help improve this manuscript. We have modified the paper in response to the extensive and insightful editor and reviewers’ comments. We have added additional explanatory items that the editor identified. Furthermore, we have revised certain sections of the manuscript and hope this addresses the referee’s remarks. We have responded to the comments point by point in the table below.

**Editor’s requirements**

|  | **Comments** | **Responses** |
| --- | --- | --- |
| 1 | A letter that responds to each point raised by the editor and reviewer(s). You should upload this letter as a separate file labeled 'Response to Reviewers'. | Thank you for your time and valuable comments. A separate letter that responds to each point raised by the editor and reviewers is prepared and uploaded, labeled ‘Response to Reviewers’. |
| 2 | A marked-up copy of your manuscript that highlights changes made to the original version. You should upload this as a separate file labeled 'Revised Manuscript with Track Changes'. | Thank you for your time and valuable comments. A separate marked-up manuscript, labeled ‘Revised Manuscript with Track Changes’, that highlights changes made to the original version and is uploaded for your review. |
| 3 | An unmarked version of your revised paper without tracked changes. You should upload this as a separate file labeled 'Manuscript'. | Thank you for your time and valuable comments. A separate marked-up manuscript, labeled ‘Manuscript’, has been uploaded for your review. |
| 4 | Guidelines for resubmitting your figure files are available below the reviewer comments at the end of this letter. | Thank you for your time and valuable comments. |
| 5 | As the corresponding author, your ORCID iD is verified in the submission system and will appear in the published article. PLOS supports the use of ORCID, and we encourage all coauthors to register for an ORCID iD and use it as well. Please encourage your coauthors to verify their ORCID iD within the submission system before final acceptance, as unverified ORCID iDs will not appear in the published article. *Only* the individual author can complete the verification step; PLOS staff *cannot* verify ORCID iDs on behalf of authors | Thank you for your time and valuable comments. All authors have verified their ORCID iD on their own as per the guidelines. |

**Journal requirements**

|  | **Comments** | **Responses** |
| --- | --- | --- |
| 1 | If the authors have adequately addressed your comments raised in a previous round of review and you feel that this manuscript is now acceptable for publication, you may indicate that here to bypass the “Comments to the Author” section, enter your conflict of interest statement in the “Confidential to Editor” section, and submit your "Accept" recommendation.  Reviewer #2: All comments have been addressed  Reviewer #4: (No Response) | Thank you for your time and valuable comments. |
| 2 | Does this manuscript meet PLOS Global Public Health’s [publication criteria](https://track.editorialmanager.com/CL0/https:%2F%2Fjournals.plos.org%2Fglobalpublichealth%2Fs%2Fcriteria-for-publication/1/010f019fd4d44d4a-160c0c15-da63-4923-9018-05422ba5540f-000000/_L-sRrStcT7Wdwhy9s3xukM1WNdFklX4yJyf9e-Dj_M=258)? Is the manuscript technically sound, and do the data support the conclusions? The manuscript must describe methodologically and ethically rigorous research with conclusions that are appropriately drawn based on the data presented.  Reviewer #2: Yes  Reviewer #4: Yes | Thank you for your time and valuable comments. |
| 3 | Has the statistical analysis been performed appropriately and rigorously?  Reviewer #2: Yes  Reviewer #4: Yes | Thank you for your time and valuable comments. |
| 4 | Have the authors made all data underlying the findings in their manuscript fully available (please refer to the Data Availability Statement at the start of the manuscript PDF file)?  The [PLOS Data policy](https://track.editorialmanager.com/CL0/https:%2F%2Fjournals.plos.org%2Fglobalpublichealth%2Fs%2Fdata-availability/1/010f019fd4d44d4a-160c0c15-da63-4923-9018-05422ba5540f-000000/QJEQB6ub06PYVisetySKLDoRpmdsHYdbkvVgG03NmkU=258) requires authors to make all data underlying the findings described in their manuscript fully available without restriction, with rare exception. The data should be provided as part of the manuscript or its supporting information, or deposited to a public repository. For example, in addition to summary statistics, the data points behind means, medians and variance measures should be available. If there are restrictions on publicly sharing data—e.g. participant privacy or use of data from a third party—those must be specified.  Reviewer #2: Yes  Reviewer #4: Yes | Thank you for your time and valuable comments. |
| 5 | Is the manuscript presented in an intelligible fashion and written in standard English?  PLOS Global Public Health does not copyedit accepted manuscripts, so the language in submitted articles must be clear, correct, and unambiguous. Any typographical or grammatical errors should be corrected at revision, so please note any specific errors here.  Reviewer #2: Yes  Reviewer #4: No | Thank you for your time and valuable comments. We have revised the manuscript and improved the document by writing in standard English; thus, it is clear, correct, and well understood, and the grammar has been improved. It is labeled ‘Manuscript’ and uploaded for your review. |
| 6 | **Review Comments to the Author**  Please use the space provided to explain your answers to the questions above. You may also include additional comments for the author, including concerns about dual publication, research ethics, or publication ethics. (Please upload your review as an attachment if it exceeds 20,000 characters)  Reviewer #2: Thank you for responding to my comments! | Thank you for your time and valuable comments. |
| 6a | Reviewer #4: Hi, thanks for the current revision and addressing some of the comments. Only minor edits are needed, as in the last revision there are errors that could easily be edited such as: * Abstract - Introduction section: the 3rd sentence ("Aims to add depth...") does not have a subject. * Order of authors in the abstract is not the same as in the manuscript. * Please check that all Anopheles are written accordingly. * Please ensure that all tables and figures are written accordingly, with the same font and sizes, and the same goes when referring them in sentences. * Not all abbreviations are listed in the abbreviation list. * For structure, consider moving the 'Ethics statement' and 'Consent for publication' into the Declaration section, instead of the current 'Materials and Implementation' section. * Please ensure that the references, are written as per journal format, such as #54.  Otherwise, I have no further comment. Thank you and best wishes. | Thank you for your time and valuable comments. We have revised the manuscript and edited it properly by;  Improving the abstract from the 3^rd^ sentence and the general abstract. Also, we have aligned the author sequence in the abstract; it is now the same as in the manuscript. Besides, we have checked the word "Anopheles" and written it accordingly, revised all the tables and figures to use the proper font and sizes, and added more abbreviations that were not listed.  Also, we have moved the ‘Ethics statements’ and ‘Consent for publication’ into the Declaration section.  Furthermore, we have improved all references by formatting them in accordance with the journal's style.  The revised manuscript, labeled ‘manuscript with Track-changes’, and the clean manuscript, labeled ‘Manuscript’, were both uploaded for your review. |
| 7 | 7. PLOS authors have the option to publish the peer review history of their article ([what does this mean?](https://track.editorialmanager.com/CL0/https:%2F%2Fjournals.plos.org%2Fglobalpublichealth%2Fs%2Feditorial-and-peer-review-process%23loc-peer-review-history/1/010f019fd4d44d4a-160c0c15-da63-4923-9018-05422ba5540f-000000/bp0DQNZodn_DcYFwCM2pyG0wmWbZjstu9w2FMnZ9qRw=258)). If published, this will include your full peer review and any attached files.   **Do you want your identity to be public for this peer review?** If you choose “no”, your identity will remain anonymous but your review may still be made public.   For information about this choice, including consent withdrawal, please see our [Privacy Policy](https://track.editorialmanager.com/CL0/https:%2F%2Fwww.plos.org%2Fprivacy-policy/1/010f019fd4d44d4a-160c0c15-da63-4923-9018-05422ba5540f-000000/Y4VGdr3LE1tda0zOAADRf1o95bSy3mhvAKv89DAcdJE=258).  Reviewer #2: No  Reviewer #4: No | Thank you for your time and valuable comments. |

We look forward to hearing from you regarding our submission. We would be happy to respond to any further questions or comments you may have. Thank you once again for your most valuable time and input.

Kind regards

Mponeja Gitanya, BSc, MPH

Corresponding author

Principal Investigator: **Strategies and approaches to vector control effectiveness towards malaria elimination in mainland Tanzania: An implementation case study analysis**

KCMC University, Moshi, Kilimanjaro, Tanzania

P.O.BOX 2240, Moshi,

Kilimanjaro, Tanzania

Mob: +55 713 719909
