# Supplementary material for: Strategies and approaches to vector control effectiveness toward the elimination of malaria in mainland Tanzania: An implementation case study analysis
Source: PLOS Glob Public Health. 2026 Oct 22;6(10):e0005574. doi: 10.1371/journal.pgph.0005574 (PMC13690203; doi:10.1371/journal.pgph.0005574)
Supplement: Supplementary file 6 [file pgph.0005574.s007.docx]

03^rd^ October 2026

**Re: PLOS Global Public Health Decision: Revision required [PGPH-D-25-02234R4] - [EMID: defb9214d48fde94]**

**PGPH-D-25-02234R4
Strategies and approaches to vector control effectiveness towards malaria elimination in mainland Tanzania: An implementation case study analysis
PLOS Global Public Health**

Dear

Kanokwan Suwannarong, Ph.D

Academic Editor
PLOS Global Public Health

Thank you for taking the time to consider our manuscript for publication in PLOS Global Public Health Journal and for providing your valuable comments.

We appreciate your input, which will help improve this manuscript. We have revised the paper in response to the editor's and reviewers’ extensive and insightful comments. We have added additional explanatory items that the editor identified. We also revised certain sections of the manuscript and hope this addresses the referee’s remarks. We have responded to the comments point by point in the table below.

**Editor’s requirements**

|  | **Comments** | **Responses** |
| --- | --- | --- |
| 1 | A letter that responds to each point raised by the editor and reviewer(s). You should upload this letter as a separate file labeled 'Response to Reviewers'. | Thank you for your time and valuable comments. A separate letter that responds to each point raised by the editor and reviewers is prepared and uploaded, labeled ‘Response to Reviewers’. |
| 2 | A marked-up copy of your manuscript that highlights changes made to the original version. You should upload this as a separate file labeled 'Revised Manuscript with Track Changes'. | Thank you for your time and valuable comments. A separate marked-up manuscript, labeled ‘Revised Manuscript with Track Changes’, that highlights changes made to the original version and is uploaded for your review. |
| 3 | An unmarked version of your revised paper without tracked changes. You should upload this as a separate file labeled 'Manuscript'. | Thank you for your time and valuable comments. A separate marked-up manuscript, labeled ‘Manuscript’, has been uploaded for your review. |
| 4 | Guidelines for resubmitting your figure files are available below the reviewer comments at the end of this letter. | Thank you for your time and valuable comments. |
| 5 | As the corresponding author, your ORCID iD is verified in the submission system and will appear in the published article. PLOS supports the use of ORCID, and we encourage all coauthors to register for an ORCID iD and use it as well. Please encourage your coauthors to verify their ORCID iD within the submission system before final acceptance, as unverified ORCID iDs will not appear in the published article. *Only* the individual author can complete the verification step; PLOS staff *cannot* verify ORCID iDs on behalf of authors | Thank you for your time and valuable comments. All authors have verified their ORCID iD on their own as per the guidelines. |

**Additional Editor Comments:**

|  | **Comments** | **Responses** |
| --- | --- | --- |
| 1 | **Abstract:** Please manually review the entire Abstract to ensure that all sentences are grammatically correct. The reviewer noted that there is still a sentence without a subject in the first paragraph | Thank you for your time and valuable comments. We conducted a thorough manual review of the Abstract and revised several sentences to improve grammar, clarity, and overall readability. |
| 2 | **Manuscript:** Please manually check all *Anopheles* species names throughout the manuscript to ensure that they are written accurately and consistently. Please also review all figure headings and ensure that they follow the journal’s required format and standards. | Thank you for your time and valuable comments. We reviewed all Anopheles species names throughout the manuscript to ensure accurate and consistent nomenclature. |
| 3 | **References:** Please carefully check each reference manually to ensure full compliance with the journal’s reference style and formatting requirements. The reviewer specifically identified errors in References #6 and #71, but the entire reference list should be checked for similar issues | Thank you for your time and valuable comments. We reviewed the figure headings and captions and made sure they comply with the journal's formatting requirements. Besides, we carefully checked and corrected the reference list, including the references specifically highlighted by the reviewer, References #6 and #71, and reviewed the entire bibliography for consistency with the journal’s reference style |

We believe that these revisions have substantially improved the manuscript and addressed all concerns raised during the review process. We respectfully submit the revised manuscript for your consideration and hope it is now suitable for publication in PLOS Global Public Health.

Thank you once again for your time and consideration. We look forward to hearing from you.

Sincerely,

Mponeja Gitanya, BSc, MPH

Corresponding author

Principal Investigator: **Strategies and approaches to vector control effectiveness towards malaria elimination in mainland Tanzania: An implementation case study analysis**

KCMC University, Moshi, Kilimanjaro, Tanzania

P.O.BOX 2240, Moshi,

Kilimanjaro, Tanzania

Mob: +55 713 719909
