# Supplementary material for: Strategies and approaches to vector control effectiveness toward the elimination of malaria in mainland Tanzania: An implementation case study analysis
Source: PLOS Glob Public Health. 2026 Oct 22;6(10):e0005574. doi: 10.1371/journal.pgph.0005574 (PMC13690203; doi:10.1371/journal.pgph.0005574)
Supplement: Peer review history

# Peer review: Decision Letter 0

**DOI:** 10.1371/journal.pgph.0005574.r001

**Authors:** Akim Tafadzwa Lukwa

3 Oct 2025

PGPH-D-25-02234

Strategies and approaches to vector control effectiveness towards malaria elimination in mainland Tanzania: An implementation case study analysis

PLOS Global Public Health

Dear Dr. Mponeja,

Thank you for submitting your manuscript to PLOS Global Public Health. After careful consideration, we feel that it has merit but does not fully meet PLOS Global Public Health’s publication criteria as it currently stands. Therefore, we invite you to submit a revised version of the manuscript that addresses the points raised during the review process.

Please submit your revised manuscript by 28 October 2025. If you will need more time than this to complete your revisions, please reply to this message or contact the journal office at globalpubhealth@plos.org. When you're ready to submit your revision, log on to https://www.editorialmanager.com/pgph/ and select the 'Submissions Needing Revision' folder to locate your manuscript file.

Please include the following items when submitting your revised manuscript:

- A rebuttal letter that responds to each point raised by the editor and reviewer(s). You should upload this letter as a separate file labeled 'Response to Reviewers'.
- A marked-up copy of your manuscript that highlights changes made to the original version. You should upload this as a separate file labeled 'Revised Manuscript with Track Changes'.
- An unmarked version of your revised paper without tracked changes. You should upload this as a separate file labeled 'Manuscript'.

Guidelines for resubmitting your figure files are available below the reviewer comments at the end of this letter.

We look forward to receiving your revised manuscript.

Kind regards,

Akim Tafadzwa Lukwa, Ph.D Public Health- Health Economics

Academic Editor

PLOS Global Public Health

Journal Requirements:

1. Please provide a detailed online Financial Disclosure statement. This is published with the article. It must therefore be completed in full sentences and contain the exact wording you wish to be published.

a) State the initials, alongside each funding source, of each author to receive each grant, if applicable. For example: “This work was supported by the National Institutes of Health (####### to AM; ###### to CJ) and the National Science Foundation (###### to AM).”

b) State what role the funders took in the study. If the funders had no role in your study, please state: “The funders had no role in study design, data collection and analysis, decision to publish, or preparation of the manuscript.”

For more information, please see our guidelines: https://journals.plos.org/globalpublichealth/s/submission-guidelines#loc-financial-disclosure-statement

2. Please ensure that the funders and grant numbers match between the Financial Disclosure field and the Funding Information tab in your submission form. Note that the funders must be provided in the same order in both places as well.

3. Please update your online Competing Interests statement. If you have no competing interests to declare, please state: “The authors have declared that no competing interests exist.”

4. For studies involving third-party data, we encourage authors to share any data specific to their analyses that they can legally distribute. PLOS recognizes, however, that authors may be using third-party data they do not have the rights to share. When third-party data cannot be publicly shared, authors must provide all information necessary for interested researchers to apply to gain access to the data. (https://journals.plos.org/globalpublichealth/s/data-availability#loc-acceptable-data-access-restrictions)

For any third-party data that the authors cannot legally distribute, they should include the following information in their Data Availability Statement upon submission:

a) A description of the data set and the third-party source

b) If applicable, verification of permission to use the data set

c) Confirmation of whether the authors received any special privileges in accessing the data that other researchers would not have

d) All necessary contact information others would need to apply to gain access to the data.

5. Some material included in your submission may be copyrighted. According to PLOS’s copyright policy, authors who use figures or other material (e.g., graphics, clipart, maps) from another author or copyright holder must demonstrate or obtain permission to publish this material under the Creative Commons Attribution 4.0 International (CC BY 4.0) License used by PLOS journals. Please closely review the details of PLOS’s copyright requirements here: PLOS Licenses and Copyright. If you need to request permissions from a copyright holder, you may use PLOS's Copyright Content Permission form.

Please respond directly to this email or email the journal office and provide any known details concerning your material's license terms and permissions required for reuse, even if you have not yet obtained copyright permissions or are unsure of your material's copyright compatibility.

Potential Copyright Issues:

Figures 4, 8 and S1 Figure: please (a) provide a direct link to the base layer of the map (i.e., the country or region border shape) and ensure this is also included in the figure legend; and (b) provide a link to the terms of use / license information for the base layer image or shapefile. We cannot publish proprietary or copyrighted maps (e.g. Google Maps, Mapquest) and the terms of use for your map base layer must be compatible with our CC-BY 4.0 license.

Note: if you created the map in a software program like R or ArcGIS, please locate and indicate the source of the basemap shapefile onto which data has been plotted.

If your map was obtained from a copyrighted source please amend the figure so that the base map used is from an openly available source. Alternatively, please provide explicit written permission from the copyright holder granting you the right to publish the material under our CC-BY 4.0 license.

Please note that the following CC BY licenses are compatible with PLOS license: CC BY 4.0, CC BY 2.0 and CC BY 3.0, meanwhile such licenses as CC BY-ND 3.0 and others are not compatible due to additional restrictions.

If you are unsure whether you can use a map or not, please do reach out and we will be able to help you. The following websites are good examples of where you can source open access or public domain maps:

* U.S. Geological Survey (USGS) - All maps are in the public domain. (http://www.usgs.gov)

* PlaniGlobe - All maps are published under a Creative Commons license so please cite “PlaniGlobe, http://www.planiglobe.com, CC BY 2.0” in the image credit after the caption. (http://www.planiglobe.com/?lang=enl)

* Natural Earth - All maps are public domain. (http://www.naturalearthdata.com/about/terms-of-use/)

6. If the reviewer comments include a recommendation to cite specific previously published works, please review and evaluate these publications to determine whether they are relevant and should be cited. There is no requirement to cite these works unless the editor has indicated otherwise.

Additional Editor Comments (if provided):

Thank you for your submission to PLOS Global Public Health. Your manuscript presents a valuable case study of malaria vector control in mainland Tanzania, with strengths in its clear presentation, thoughtful classification of councils by transmission intensity, and integration of multiple data sources. This type of detailed implementation analysis is rare and important for informing malaria elimination strategies.

That said, both reviewers and the editorial team feel that the paper requires major revision before it can be considered further. In particular, please consider the following points:

Contextualization and Literature Review

-Expand the introduction to provide a regional perspective, situating Tanzania’s experiences within the broader East African and African malaria control landscape.

-Incorporate discussion of regional malaria interventions and how Tanzania’s strategies align with or differ from neighbouring countries.

-Explicitly integrate the role of climate change and climatic variability in shaping malaria transmission patterns and vector control effectiveness.

Climate and Future Projections

-The results and discussion would benefit from incorporating or at least discussing climate projections (e.g., rainfall, temperature trends) and their potential impacts on malaria incidence and vector behaviour.

-Even if primary modelling is not feasible, a narrative integration of existing projections could strengthen the “forward-looking” contribution of the study.

Methods Clarification

-Provide additional details on the modelling assumptions for ITN survival and decay, especially given regional variations.

-Clarify how rainfall and seasonality data were linked to malaria incidence, and whether projections or anomalies were considered.

Discussion and Policy Implications

-Expand the discussion to link your findings more explicitly with policy implications at regional (East Africa) and continental (Africa-wide) levels.

-Consider highlighting how lessons from Tanzania might inform other malaria-endemic countries with similar ecological and epidemiological settings.

Language and Presentation

-While the manuscript is overall clear, a careful language edit will help tighten some sections and improve readability.

-Figures and tables are informative, but please ensure they are high-resolution and formatted according to journal standards.

In summary, your manuscript has significant potential, but revisions are needed to strengthen its contextual relevance, integrate climate considerations, and extend its contribution beyond Tanzania. We encourage you to resubmit after addressing the above points.

[Note: HTML markup is below. Please do not edit.]

Reviewers' comments:

Reviewer's Responses to Questions

**Comments to the Author**

1. Does this manuscript meet PLOS Global Public Health’s publication criteria?

Reviewer #1: Yes

Reviewer #2: Yes

**********

2. Has the statistical analysis been performed appropriately and rigorously?

Reviewer #1: Yes

Reviewer #2: No

**********

3. Have the authors made all data underlying the findings in their manuscript fully available (please refer to the Data Availability Statement at the start of the manuscript PDF file)?

The PLOS Data policy

Reviewer #1: Yes

Reviewer #2: Yes

**********

4. Is the manuscript presented in an intelligible fashion and written in standard English?

Reviewer #1: Yes

Reviewer #2: Yes

**********

Reviewer #1: Good analysis of data and interpretation. Rare publication that supports vector control in Africa. Cases were discussed in relation to vector control methods, availability of malaria vectors and their response to insecticides used for their control. classification of transmission areas on the basis of transmission levels and rainfall patterns is good. The manuscript is presented in a way that can be understood. The English language used in the manuscript can be comprehended. Writing in the manuscript is clear and correct.

Reviewer #2: Thak you very much for inviting me to review this paper!

While the authors have attempted to paint a picture of the problem within Tanzania, I feel the paper would benefit with a review of the malaria interventions within East Africa and integrate aspects of climate change. This could also be done at regional level (Africa). I feel doing this in the introduction will improve understanding of the problem.

For the methods and results, can the authors also do some projections on climate variables and malaria? I feel this will also improve the paper by providing a probable future perspective.

**********

what does this mean?). If published, this will include your full peer review and any attached files.

**Do you want your identity to be public for this peer review?**  If you choose “no”, your identity will remain anonymous but your review may still be made public.

For information about this choice, including consent withdrawal, please see our Privacy Policy

Reviewer #1: **Yes:**  Nzira Lukwa

Reviewer #2: No

**********

[NOTE: If reviewer comments were submitted as an attachment file, they will be attached to this email and accessible via the submission site. Please log into your account, locate the manuscript record, and check for the action link "View Attachments". If this link does not appear, there are no attachment files.]

# Author comment: Author response to Decision Letter 1

**DOI:** 10.1371/journal.pgph.0005574.r002

2 Dec 2025

Attachment Submitted filename: Response-Reviewers-comments PLOS- 03Dec2025.pdf

# Peer review: Decision Letter 1

**DOI:** 10.1371/journal.pgph.0005574.r003

**Authors:** Miquel Vall-llosera Camps

16 Feb 2026

PGPH-D-25-02234R1

Strategies and approaches to vector control effectiveness towards malaria elimination in mainland Tanzania: An implementation case study analysis

PLOS Global Public Health

Dear Dr. Gitanya,

Thank you for submitting your manuscript to PLOS Global Public Health. After careful consideration, we feel that it has merit but does not fully meet PLOS Global Public Health’s publication criteria as it currently stands. Therefore, we invite you to submit a revised version of the manuscript that addresses the points raised during the review process.

Please submit your revised manuscript by Mar 18 2026 11:59PM. If you will need more time than this to complete your revisions, please reply to this message or contact the journal office at globalpubhealth@plos.org. When you're ready to submit your revision, log on to https://www.editorialmanager.com/pgph/ and select the 'Submissions Needing Revision' folder to locate your manuscript file.

Please include the following items when submitting your revised manuscript:

- A letter that responds to each point raised by the editor and reviewer(s). You should upload this letter as a separate file labeled 'Response to Reviewers'.
- A marked-up copy of your manuscript that highlights changes made to the original version. You should upload this as a separate file labeled 'Revised Manuscript with Track Changes'.
- An unmarked version of your revised paper without tracked changes. You should upload this as a separate file labeled 'Manuscript'.

Guidelines for resubmitting your figure files are available below the reviewer comments at the end of this letter.

We look forward to receiving your revised manuscript.

Kind regards,

Miquel Vall-llosera Camps

Staff Editor

PLOS Global Public Health

Journal Requirements:

If the reviewer comments include a recommendation to cite specific previously published works, please review and evaluate these publications to determine whether they are relevant and should be cited. There is no requirement to cite these works unless the editor has indicated otherwise.

Please review your reference list to ensure that it is complete and correct. If you have cited papers that have been retracted, please include the rationale for doing so in the manuscript text, or remove these references and replace them with relevant current references. Any changes to the reference list should be mentioned in the rebuttal letter that accompanies your revised manuscript. If you need to cite a retracted article, indicate the article’s retracted status in the References list and also include a citation and full reference for the retraction notice.

[Note: HTML markup is below. Please do not edit.]

Reviewers' comments:

Reviewer's Responses to Questions

**Comments to the Author**

Reviewer #3: All comments have been addressed

**********

publication criteria?

Reviewer #3: Yes

**********

3. Has the statistical analysis been performed appropriately and rigorously?

Reviewer #3: Yes

**********

4. Have the authors made all data underlying the findings in their manuscript fully available (please refer to the Data Availability Statement at the start of the manuscript PDF file)?

The PLOS Data policy

Reviewer #3: No

**********

5. Is the manuscript presented in an intelligible fashion and written in standard English?

Reviewer #3: Yes

**********

Reviewer #3: Overall, this is a very useful case report with methods sure to have future impact. Thank you for compiling this.

Figure 1: Number of nets hard to relate to coverage without size of population. Similarly to the black line in figure 4, consider adding a line which is the number of nets needed each year to reach 80% coverage given median net life of 2.15 years.

To me a logical order for the methods section would be to put the malaria case incidence first. A proposed order would be: Malaria case incidence analysis, Insecticide-treated net data sources, Entomological surveillance and insecticide resistance, Rainfall and Seasonality and Council classification framework.

Figure 2: There is no mention of how PBO nets are quantified in the methods. The last methods section reads “PBO nets were advised in councils with pyrethroid resistance, provided budget constraints didn’t compromise overall ITN coverage”. However, Figure 2 has quantified this. Please explain this in the methods.

Figure 5: It is unclear from text or figure if linear regressions showed a significant trend.

Results – Vector species composition. “Other vector distributions are shown below” Where? Table 2? I cannot see this referenced in the text. Please change “below” to “Table 2”. Also, species composition is not mentioned in the table legend.

Finally, a few of the figure axis labels and legends and tables text are appearing blurry in the complied PDF. Please make sure the quality of these is in accordance to journal specifications.

Please make your data and estimations available as supplementary data for potential future analysis. While plots are shown, these pool the estimated net coverage into <50%, 50-80% and >80%, the actual estimated values are not reported. For example a table with the data from figure 9 with cases each year additionally would significantly increase the usefulness for potential future analysis. I hope you make resources openly available beyond DHIS 2.

**********

what does this mean?). If published, this will include your full peer review and any attached files.

**Do you want your identity to be public for this peer review?**  If you choose “no”, your identity will remain anonymous but your review may still be made public.

For information about this choice, including consent withdrawal, please see our Privacy Policy

Reviewer #3: **Yes:**  Emma Louise Fairbanks

**********

[NOTE: If reviewer comments were submitted as an attachment file, they will be attached to this email and accessible via the submission site. Please log into your account, locate the manuscript record, and check for the action link "View Attachments". If this link does not appear, there are no attachment files.]

# Author comment: Author response to Decision Letter 2

**DOI:** 10.1371/journal.pgph.0005574.r004

17 Mar 2026

Attachment Submitted filename: Response to-Reviewers-1732026.docx

# Peer review: Decision Letter 2

**DOI:** 10.1371/journal.pgph.0005574.r005

**Authors:** Kanokwan Suwannarong

23 Apr 2026

PGPH-D-25-02234R2

Strategies and approaches to vector control effectiveness towards malaria elimination in mainland Tanzania: An implementation case study analysis

PLOS Global Public Health

Dear Dr. Gitanya,

Thank you for submitting your manuscript to PLOS Global Public Health. After careful consideration, we feel that it has merit but does not fully meet PLOS Global Public Health’s publication criteria as it currently stands. Therefore, we invite you to submit a revised version of the manuscript that addresses the points raised during the review process.

Please submit your revised manuscript by May 23 2026 11:59PM. If you will need more time than this to complete your revisions, please reply to this message or contact the journal office at globalpubhealth@plos.org. When you're ready to submit your revision, log on to https://www.editorialmanager.com/pgph/ and select the 'Submissions Needing Revision' folder to locate your manuscript file.

Please include the following items when submitting your revised manuscript:

- A letter that responds to each point raised by the editor and reviewer(s). You should upload this letter as a separate file labeled 'Response to Reviewers'.
- A marked-up copy of your manuscript that highlights changes made to the original version. You should upload this as a separate file labeled 'Revised Manuscript with Track Changes'.
- An unmarked version of your revised paper without tracked changes. You should upload this as a separate file labeled 'Manuscript'.

Guidelines for resubmitting your figure files are available below the reviewer comments at the end of this letter.

As the corresponding author, your ORCID iD is verified in the submission system and will appear in the published article. PLOS supports the use of ORCID, and we encourage all coauthors to register for an ORCID iD and use it as well. Please encourage your coauthors to verify their ORCID iD within the submission system before final acceptance, as unverified ORCID iDs will not appear in the published article. *Only*  the individual author can complete the verification step; PLOS staff *cannot*  verify ORCID iDs on behalf of authors.

We look forward to receiving your revised manuscript.

Kind regards,

Kanokwan Suwannarong, Ph.D.

Academic Editor

PLOS Global Public Health

**Journal Requirements:**

If the reviewer comments include a recommendation to cite specific previously published works, please review and evaluate these publications to determine whether they are relevant and should be cited. There is no requirement to cite these works unless the editor has indicated otherwise.

Please review your reference list to ensure that it is complete and correct. If you have cited papers that have been retracted, please include the rationale for doing so in the manuscript text, or remove these references and replace them with relevant current references. Any changes to the reference list should be mentioned in the rebuttal letter that accompanies your revised manuscript. If you need to cite a retracted article, indicate the article’s retracted status in the References list and also include a citation and full reference for the retraction notice.

**Reviewers' comments:**

Reviewer's Responses to Questions

**Comments to the Author**

Reviewer #3: All comments have been addressed

Reviewer #4: All comments have been addressed

Reviewer #5: (No Response)

**********

publication criteria?

Reviewer #3: Yes

Reviewer #4: Yes

Reviewer #5: Yes

**********

3. Has the statistical analysis been performed appropriately and rigorously?

Reviewer #3: Yes

Reviewer #4: Yes

Reviewer #5: Yes

**********

4. Have the authors made all data underlying the findings in their manuscript fully available (please refer to the Data Availability Statement at the start of the manuscript PDF file)?

The PLOS Data policy

Reviewer #3: Yes

Reviewer #4: Yes

Reviewer #5: Yes

**********

5. Is the manuscript presented in an intelligible fashion and written in standard English?

Reviewer #3: Yes

Reviewer #4: No

Reviewer #5: Yes

**********

**Reviewer #3:**  (No Response)

**Reviewer #4:**  The study provides a useful approach in analysing vector control distribution and access in selected regions where interventions and data were fragmented, by integrating data of intervention coverage from various interventions, which would be useful for malaria program and decision makers in Tanzania, as well as globally.

Strengths:

The writers have put a lot of effort in comprehensively combined multiple data sources from various intervention coverages and presented them at a council level to provide recommendation for the decision makers for a more tailored program planning.

Things to improve:

If there is any recommendation (such as for data segregation and analysis) regarding changes in council boundaries/designation overtime, as well as population shifts at subnational level, consider adding them in the recommendation section, whenever possible.

With the useful compilation and complex analysis of various data sources, clarity is important, particularly for decision makers and program replicability. An example can be seen on the 'Vector control coverage' of the 'Result' section, the first paragraph discussed about IRS in several regions, while the second paragraph directly started with “For example …” and then discussed about ITN distribution, with minimal explanation that link both paragraphs. Consider rephrasing for improved writing structure as this manuscript might be useful for malaria program.

While the manuscript is overall clear, a language edit would improve clarity and readability. These are minor but would be an important edit that include:

• Proper use of punctuations (such as in some abbreviations of Anopheles) and grammar, where a few sentences can be found without subjects (e. g. the two aims in the ‘Abstract – Introduction’ section and the method in ‘ITN data sources’ section on pp. 7-8); while some sentences without verbs simply due to misplaced of punctuations (e. g. a few sentences on the first paragraph of ‘Materials and implementation methods’ section).

• Generally, it is recommended to spell out the full term upon first mention, followed by abbreviation in parentheses, then use only the abbreviation afterwards, and to include all relevant abbreviations in the abbreviation list.

Additional recommendations on data in figures and tables for consideration:

• Headings of figures and tables should include concise, yet accurate information, such area, timeline, reference the data source, if applicable

• Figures/Tables should be written as per journal format

• To show clear information on Legend and avoid overlapping with the image, such as in Figure 9.

• Be specific for clarity and include the table/figure number when referring within a sentence. For example, in p. 8, the Rainfall and Seasonality section (… presented as overlapping monthly time series relative to malaria case incidence trends [as shown in …]); or in p. 14 (“Other vector distributions are shown [in table 2] below.”)

Other points:

For structure, I would recommend repositioning the 'Ethics statement' and 'Consent for publication' from the ‘Materials and Implementation method’ section into the ‘Declaration’ section.

Minor edit would be required for some reference links that are not valid and references that not written as per journal format.

Thank you for the compilation and best wishes.

**Reviewer #5:**  well done research manuscript.

**********

what does this mean?). If published, this will include your full peer review and any attached files.

**Do you want your identity to be public for this peer review?**  If you choose “no”, your identity will remain anonymous but your review may still be made public.

For information about this choice, including consent withdrawal, please see our Privacy Policy

Reviewer #3: **Yes:**  Emma Fairbanks

Reviewer #4: No

Reviewer #5: **Yes:**  Guta Gurmessa Amenu

**********

[NOTE: If reviewer comments were submitted as an attachment file, they will be attached to this email and accessible via the submission site. Please log into your account, locate the manuscript record, and check for the action link "View Attachments". If this link does not appear, there are no attachment files.]

**Figure Resubmissions:**

# Author comment: Author response to Decision Letter 3

**DOI:** 10.1371/journal.pgph.0005574.r006

22 Jun 2026

Attachment Submitted filename: Response to-Reviewers-02June2026.docx

# Peer review: Decision Letter 3

**DOI:** 10.1371/journal.pgph.0005574.r007

**Authors:** Kanokwan Suwannarong

5 Aug 2026

PGPH-D-25-02234R3

Strategies and approaches to vector control effectiveness toward malaria elimination in mainland Tanzania: An implementation case study analysis

PLOS Global Public Health

Dear Dr. Gitanya,

Thank you for submitting your manuscript to PLOS Global Public Health. After careful consideration, we feel that it has merit but does not fully meet PLOS Global Public Health’s publication criteria as it currently stands. Therefore, we invite you to submit a revised version of the manuscript that addresses the points raised during the review process.

**Kindly review the reviewers' comments carefully and revise your manuscript accordingly to improve its quality.**

Please submit your revised manuscript by Sep 04 2026 11:59PM. If you will need more time than this to complete your revisions, please reply to this message or contact the journal office at globalpubhealth@plos.org. When you're ready to submit your revision, log on to https://www.editorialmanager.com/pgph/ and select the 'Submissions Needing Revision' folder to locate your manuscript file.

Please include the following items when submitting your revised manuscript:

- A letter that responds to each point raised by the editor and reviewer(s). You should upload this letter as a separate file labeled 'Response to Reviewers'.
- A marked-up copy of your manuscript that highlights changes made to the original version. You should upload this as a separate file labeled 'Revised Manuscript with Track Changes'.
- An unmarked version of your revised paper without tracked changes. You should upload this as a separate file labeled 'Manuscript'.

Guidelines for resubmitting your figure files are available below the reviewer comments at the end of this letter.

As the corresponding author, your ORCID iD is verified in the submission system and will appear in the published article. PLOS supports the use of ORCID, and we encourage all coauthors to register for an ORCID iD and use it as well. Please encourage your coauthors to verify their ORCID iD within the submission system before final acceptance, as unverified ORCID iDs will not appear in the published article. *Only*  the individual author can complete the verification step; PLOS staff *cannot*  verify ORCID iDs on behalf of authors.

We look forward to receiving your revised manuscript.

Kind regards,

Kanokwan Suwannarong, Ph.D.

Academic Editor

PLOS Global Public Health

Journal Requirements:

If the reviewer comments include a recommendation to cite specific previously published works, please review and evaluate these publications to determine whether they are relevant and should be cited. There is no requirement to cite these works unless the editor has indicated otherwise.

Please review your reference list to ensure that it is complete and correct. If you have cited papers that have been retracted, please include the rationale for doing so in the manuscript text, or remove these references and replace them with relevant current references. Any changes to the reference list should be mentioned in the rebuttal letter that accompanies your revised manuscript. If you need to cite a retracted article, indicate the article’s retracted status in the References list and also include a citation and full reference for the retraction notice.

[Note: HTML markup is below. Please do not edit.]

Reviewers' comments:

Reviewer's Responses to Questions

**Comments to the Author**

Reviewer #2: All comments have been addressed

Reviewer #4: (No Response)

**********

publication criteria?

Reviewer #2: Yes

Reviewer #4: Yes

**********

3. Has the statistical analysis been performed appropriately and rigorously?

Reviewer #2: Yes

Reviewer #4: Yes

**********

4. Have the authors made all data underlying the findings in their manuscript fully available (please refer to the Data Availability Statement at the start of the manuscript PDF file)?

The PLOS Data policy

Reviewer #2: Yes

Reviewer #4: Yes

**********

5. Is the manuscript presented in an intelligible fashion and written in standard English?

Reviewer #2: Yes

Reviewer #4: No

**********

**Review Comments to the Author**

Reviewer #2: Thank you for responding to my comments!

Reviewer #4: Hi, thanks for the current revision and addressing some of the comments.

Only minor edits are needed, as in the last revision there are errors that could easily be edited such as:

* Abstract - Introduction section: the 3rd sentence ("Aims to add depth...") does not have a subject.

* Order of authors in the abstract is not the same as in the manuscript.

* Please check that all Anopheles are written accordingly.

* Please ensure that all tables and figures are written accordingly, with the same font and sizes, and the same goes when referring them in sentences.

* Not all abbreviations are listed in the abbreviation list.

* For structure, consider moving the 'Ethics statement' and 'Consent for publication' into the Declaration section, instead of the current 'Materials and Implementation' section.

* Please ensure that the references, are written as per journal format, such as #54.

Otherwise, I have no further comment. Thank you and best wishes.

**********

what does this mean?). If published, this will include your full peer review and any attached files.

**Do you want your identity to be public for this peer review?**  If you choose “no”, your identity will remain anonymous but your review may still be made public.

For information about this choice, including consent withdrawal, please see our Privacy Policy

Reviewer #2: No

Reviewer #4: No

**********

[NOTE: If reviewer comments were submitted as an attachment file, they will be attached to this email and accessible via the submission site. Please log into your account, locate the manuscript record, and check for the action link "View Attachments". If this link does not appear, there are no attachment files.]

# Author comment: Author response to Decision Letter 4

**DOI:** 10.1371/journal.pgph.0005574.r008

17 Aug 2026

Attachment Submitted filename: Response to-Reviewers-17August2026.docx

# Peer review: Decision Letter 4

**DOI:** 10.1371/journal.pgph.0005574.r009

**Authors:** Kanokwan Suwannarong

21 Sep 2026

PGPH-D-25-02234R4

Strategies and approaches to vector control effectiveness toward malaria elimination in mainland Tanzania: An implementation case study analysis

PLOS Global Public Health

Dear Dr. Gitanya,

Thank you for submitting your manuscript to PLOS Global Public Health. After careful consideration, we feel that it has merit but does not fully meet PLOS Global Public Health’s publication criteria as it currently stands. Therefore, we invite you to submit a revised version of the manuscript that addresses the points raised during the review process.

Please submit your revised manuscript by Oct 21 2026 11:59PM. If you will need more time than this to complete your revisions, please reply to this message or contact the journal office at globalpubhealth@plos.org. When you're ready to submit your revision, log on to https://www.editorialmanager.com/pgph/ and select the 'Submissions Needing Revision' folder to locate your manuscript file.

Please include the following items when submitting your revised manuscript:

- A letter that responds to each point raised by the editor and reviewer(s). You should upload this letter as a separate file labeled 'Response to Reviewers'.
- A marked-up copy of your manuscript that highlights changes made to the original version. You should upload this as a separate file labeled 'Revised Manuscript with Track Changes'.
- An unmarked version of your revised paper without tracked changes. You should upload this as a separate file labeled 'Manuscript'.

Guidelines for resubmitting your figure files are available below the reviewer comments at the end of this letter.

As the corresponding author, your ORCID iD is verified in the submission system and will appear in the published article. PLOS supports the use of ORCID, and we encourage all coauthors to register for an ORCID iD and use it as well. Please encourage your coauthors to verify their ORCID iD within the submission system before final acceptance, as unverified ORCID iDs will not appear in the published article. *Only*  the individual author can complete the verification step; PLOS staff *cannot*  verify ORCID iDs on behalf of authors.

We look forward to receiving your revised manuscript.

Kind regards,

Kanokwan Suwannarong, Ph.D.

Academic Editor

PLOS Global Public Health

Journal Requirements:

If the reviewer comments include a recommendation to cite specific previously published works, please review and evaluate these publications to determine whether they are relevant and should be cited. There is no requirement to cite these works unless the editor has indicated otherwise.

Please review your reference list to ensure that it is complete and correct. If you have cited papers that have been retracted, please include the rationale for doing so in the manuscript text, or remove these references and replace them with relevant current references. Any changes to the reference list should be mentioned in the rebuttal letter that accompanies your revised manuscript. If you need to cite a retracted article, indicate the article’s retracted status in the References list and also include a citation and full reference for the retraction notice.

**Additional Editor Comments:**

Kindly review and address the reviewer’s comments very carefully, particularly the following points:

1. **Abstract:**  Please manually review the entire Abstract to ensure that all sentences are grammatically correct. The reviewer noted that there is still a sentence without a subject in the first paragraph.
2. **Manuscript:**  Please manually check all *Anopheles*  species names throughout the manuscript to ensure that they are written accurately and consistently. Please also review all figure headings and ensure that they follow the journal’s required format and standards.
3. **References:**  Please carefully check each reference manually to ensure full compliance with the journal’s reference style and formatting requirements. The reviewer specifically identified errors in References #6 and #71, but the entire reference list should be checked for similar issues.

Please do not rely solely on automated grammar or formatting tools. A careful manual review of the Abstract, species names, figure headings, and all references is particularly important before resubmission.

[Note: HTML markup is below. Please do not edit.]

Reviewers' comments:

[NOTE: If reviewer comments were submitted as an attachment file, they will be attached to this email and accessible via the submission site. Please log into your account, locate the manuscript record, and check for the action link "View Attachments". If this link does not appear, there are no attachment files.]

# Author comment: Author response to Decision Letter 5

**DOI:** 10.1371/journal.pgph.0005574.r010

3 Oct 2026

Attachment Submitted filename: Response to-Reviewers-PGPH-D-25-02234R4-03October2026.docx

# Editor report: Decision Letter 5

**DOI:** 10.1371/journal.pgph.0005574.r011

**Authors:** Kanokwan Suwannarong

5 Oct 2026

Strategies and approaches to vector control effectiveness toward malaria elimination in mainland Tanzania: An implementation case study analysis

PGPH-D-25-02234R5

Dear Dr Gitanya,

We are pleased to inform you that your manuscript 'Strategies and approaches to vector control effectiveness toward malaria elimination in mainland Tanzania: An implementation case study analysis' has been provisionally accepted for publication in PLOS Global Public Health.

Before your manuscript can be formally accepted you will need to complete some formatting changes, which you will receive in a follow up email. A member of our team will be in touch with a set of requests.

Please note that your manuscript will not be scheduled for publication until you have made the required changes, so a swift response is appreciated.

IMPORTANT: The editorial review process is now complete. PLOS will only permit corrections to spelling, formatting or significant scientific errors from this point onwards. Requests for major changes, or any which affect the scientific understanding of your work, will cause delays to the publication date of your manuscript.

If your institution or institutions have a press office, please notify them about your upcoming paper to help maximize its impact. If they'll be preparing press materials, please inform our press team as soon as possible -- no later than 48 hours after receiving the formal acceptance. Your manuscript will remain under strict press embargo until 2 pm Eastern Time on the date of publication. For more information, please contact globalpubhealth@plos.org.

Thank you again for supporting Open Access publishing; we are looking forward to publishing your work in PLOS Global Public Health.

Best regards,

Kanokwan Suwannarong, Ph.D.

Academic Editor

PLOS Global Public Health

***********************************************************

Reviewer Comments (if any, and for reference):
